# A novel differentiated HuH-7 cell model to examine bile acid metabolism, transport and cholestatic hepatotoxicity

**DOI:** 10.1038/s41598-022-18174-z

**Published:** 2022-08-22

**Authors:** Chitra Saran, Dong Fu, Henry Ho, Abigail Klein, John K. Fallon, Paavo Honkakoski, Kim L. R. Brouwer

**Affiliations:** 1 Department of Pharmacology, UNC School of Medicine, University of North Carolina at Chapel Hill, Chapel Hill, NC USA; 2 Division of Pharmacotherapy and Experimental Therapeutics, UNC Eshelman School of Pharmacy, University of North Carolina, Chapel Hill, NC USA; 3 Division of Pharmacoengineering and Molecular Pharmaceutics, UNC Eshelman School of Pharmacy, University of North Carolina, Chapel Hill, NC USA; 4 School of Pharmacy, University of Eastern Finland, Kuopio, Finland

**Keywords:** Hepatology, Cell polarity, Toxicology, Biological models, Transporters

## Abstract

Hepatic cell lines serve as economical and reproducible alternatives for primary human hepatocytes. However, the utility of hepatic cell lines to examine bile acid homeostasis and cholestatic toxicity is limited due to abnormal expression and function of bile acid-metabolizing enzymes, transporters, and the absence of canalicular formation. We discovered that culturing HuH-7 human hepatoma cells with dexamethasone (DEX) and 0.5% dimethyl sulfoxide (DMSO) for two weeks, with Matrigel overlay after one week, resulted in a shorter and improved differentiation process. These culture conditions increased the expression and function of the major bile acid uptake and efflux transporters, sodium taurocholate co-transporting polypeptide (NTCP) and the bile salt export pump (BSEP), respectively, in two-week cultures of HuH-7 cells. This in vitro model was further characterized for expression and function of bile acid-metabolizing enzymes, transporters, and cellular bile acids. Differentiated HuH-7 cells displayed a marked shift in bile acid composition and induction of cytochrome P450 (CYP) 7A1, CYP8B1, CYP3A4, and bile acid-CoA: amino acid N-acyltransferase (BAAT) mRNAs compared to control. Inhibition of taurocholate uptake and excretion after a 24-h treatment with prototypical cholestatic drugs suggests that differentiated HuH-7 cells are a suitable model to examine cholestatic hepatotoxicity.

## Introduction

Human hepatocytes and hepatic cell lines are routinely used for the evaluation of hepatic drug metabolism, transport and toxicity^1^. Fresh or cryopreserved primary human hepatocytes that are cultured in a sandwich configuration between two gelled layers of extracellular matrix are considered the gold-standard to study drug disposition and are a validated in vitro model to assess drug-induced liver injury (DILI)^2^. However, primary human hepatocytes from healthy donors are a scarce resource and subject to inherent donor-to-donor variability^3^. Hence, there is a need for hepatic cell lines that can serve as surrogates to examine drug-induced hepatotoxicity. While hepatic cell lines are economical, reproducible, and easily manipulated, their aberrant bile acid metabolism and transport present a challenge when investigating bile acid-dependent DILI^4^.

The human hepatoma HuH-7 cell line was established in 1982 from a liver tumor in a 57 year-old Japanese male^5^. Gene expression profiling showed few similarities between HuH-7 cells and human hepatocytes^6,7^. However, characterization of drug transporter expression in HuH-7 cells revealed mRNA levels of farnesoid X receptor (FXR), nuclear factor erythroid 2-related factor 2 (Nrf2), and multidrug resistance-associated protein 2 (MRP2) comparable or higher than in human hepatocytes^8^. Confluent HuH-7 cells cultured over several weeks exhibited induction of numerous transcription factors, transporters, and important drug-metabolizing enzymes, including cytochrome P450 (CYP) 3A4, which is likely due to pregnane X receptor (PXR) activation^9^. In contrast, CYP3A4 induction was absent in confluent human hepatoma HepG2 cells^10^. Dinaciclib, a cyclin-dependent kinase inhibitor, and rifampicin have been used to activate PXR and induce CYP3A4 to enable drug interaction studies in confluent HuH-7 cells^11^. Confluent HuH-7 cells cultured over four weeks expressed organic anion transporting polypeptide (OATP) 1B3, OATP2B1, organic solute transporter (OST) α, and MRP4 protein^12^, while the bile acid transporters sodium taurocholate co-transporting polypeptide (NTCP) and bile salt export pump (BSEP) were absent^12^. Recently, expression, localization, and function of BSEP were restored in four-week confluent HuH-7 cell cultures with addition of dexamethasone (DEX) and Matrigel overlay. With these modifications, HuH-7 cells displayed hepatocyte-like morphology and bile canalicular-like formation^13^. DEX is a known ligand of the glucocorticoid receptor and induces the expression of constitutive androstane receptor (CAR) and PXR in human hepatocytes^14–16^. DEX regulates many proteins including BSEP^17^, NTCP^18^, sulfotransferase (SULT) 2A1^19^, and the CYP3A family of enzymes^20^. Dimethyl sulfoxide (DMSO) addition to HuH-7 cells resulted in CAR and CYP3A4 induction and enhanced cellular differentiation^21,22^. In short, HuH-7 cells retain the capacity for cell polarization and differentiation, and nuclear receptor-regulated bile acid metabolism and transport appears feasible, although, not fully characterized.

Hepatic bile acid homeostasis is mediated via multiple CYP enzymes, uridine 5'-diphospho-glucuronosyltransferases (UGTs) and transport proteins. Bile acids are synthesized from cholesterol primarily through the classical pathway (~ 90%)^23^. CYP7A1 converts cholesterol to 7α-hydroxycholesterol, followed by CYP8B1- and CYP27A1-mediated formation of cholic acid (CA) and chenodeoxycholic acid (CDCA), respectively. CYP7B1 and CYP27A1 also contribute to the formation of CA and CDCA via the alternative pathway of bile acid synthesis. These primary bile acids are conjugated to glycine or taurine by amidation to generate glyco- or tauro-CA (GCA or TCA, respectively) and glyco- or tauro-CDCA (GCDCA or TCDCA, respectively) by two enzymes, bile acid-CoA synthase (BACS) and bile acid-CoA: amino acid N-acyltransferase (BAAT)^24^. BSEP, the primary bile acid efflux transporter on the hepatic apical membrane^25^, excretes bile acids into the bile canaliculi. Secondary bile acids are synthesized by the intestinal bacteria, reabsorbed in the ileum and returned to the liver via enterohepatic circulation^23^, where NTCP primarily regulates hepatic basolateral bile acid uptake^26^. In addition, bile acids can be converted to glucuronide and sulfate metabolites mainly by UGT1A3, UGT2B4, UGT2B7, and SULT2A1^27,28^. Other hepatic proteins that contribute to bile acid transport are OATP1B1, OATP1B3, OATP2B1, MRP2, MRP3, MRP4, and OSTα/β^29^. Intracellular accumulation of hydrophobic bile acids via inhibition of BSEP causes cholestasis or impaired bile flow and is associated with DILI^30,31^.

The main objective of the present study was to improve and accelerate the differentiation process of HuH-7 cells to two weeks using DEX, DMSO and Matrigel overlay. This novel in vitro model was characterized for bile acid homeostasis and transporter expression, localization, and function and developed as a tool to investigate cholestatic hepatotoxicity.

## Results

### Differentiated HuH-7 cells form bile canalicular-like structures

**Figure 1:**
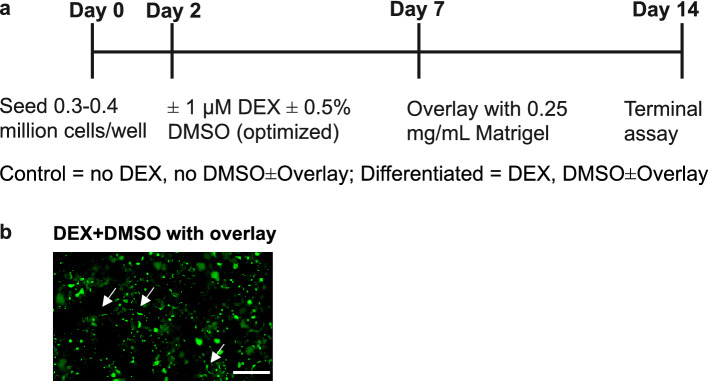
Differentiation scheme and the effect of dexamethasone (DEX) and DMSO-mediated differentiation on canalicular formation in HuH-7 cells. (**a**) Schematic depicting HuH-7 differentiation and culture timeline with 1 µM DEX and 0.5% DMSO supplementation and Matrigel overlay. Extended culture was performed for 2 weeks. (**b**) Formation of canalicular-like structures was examined using 5(6)-carboxy-2′,7′-dichlorofluorescein diacetate (CDFDA) in differentiated HuH-7 cells with overlay. Uptake of CDFDA occurs passively into the cell, where intracellular esterase enzymes cleave the diacetate group. The resulting CDF is a fluorescent substrate for apical MRP2-mediated efflux into canalicular-like structures highlighted in green (arrows). Scale bar = 200 µm.

Differentiation of HuH-7 cells with DEX and DMSO supplementation was performed using a two-week culture period as shown in Fig. 1a. Canalicular formation was confirmed in differentiated HuH-7 cells with Matrigel overlay using 5(6)-carboxy-2′,7′-dichlorofluorescein diacetate (CDFDA). CDFDA is intracellularly hydrolyzed to fluorescent CDF, which is excreted by MRP2 into bile canalicular-like networks^32^, as highlighted in Fig. 1b. This represents a morphological hallmark of polarized hepatic cells.

### Abundance of bile acid transporters and enzymes was altered in differentiated HuH-7 cells

**Figure 2:**
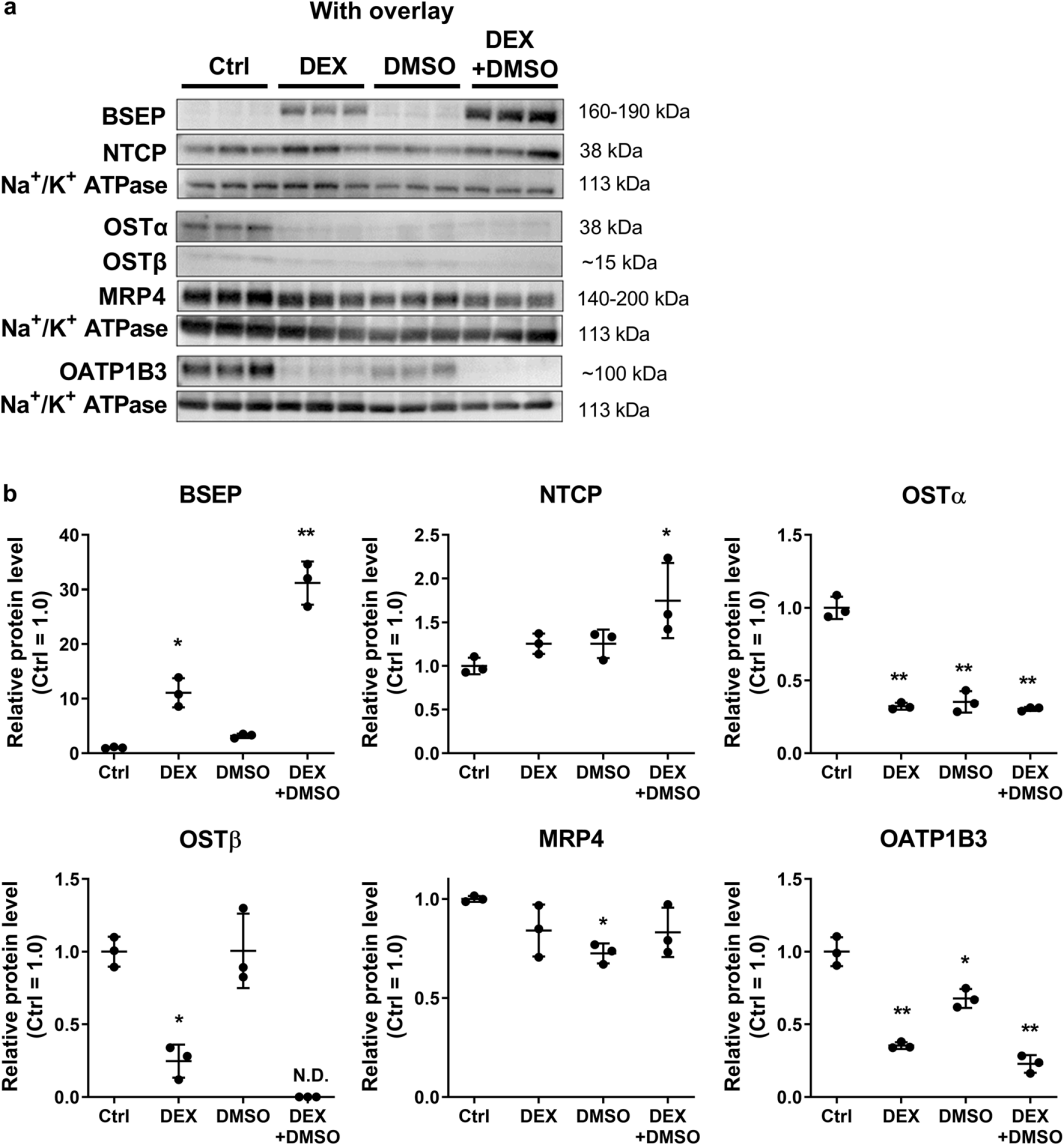
Membrane protein abundance of key hepatic bile acid transporters in control and differentiated HuH-7 cells cultured for 2 weeks with overlay. HuH-7 cells were cultured for 2 weeks without (Ctrl) and with 1 µM dexamethasone (DEX) and/or 0.5% DMSO. (**a**) Abundance of bile acid-relevant transport proteins such as the bile salt export pump (BSEP), sodium taurocholate co-transporting polypeptide (NTCP), organic solute transporter (OST) α, OST β, multidrug resistance-associated protein (MRP) 4, organic anion transporting polypeptide (OATP) 1B3, and Na^+^/K^+^ ATPase (loading control) was evaluated using Western blotting of membrane fractions harvested from HuH-7 cells with overlay. (**b**) Densitometry was performed using ImageJ and BSEP, NTCP, OSTα, OSTβ, MRP4, and OATP1B3 signals were normalized to Na^+^/K^+^ ATPase. Relative protein levels were calculated with respect to Ctrl (set to 1.0) and data were plotted as mean ± standard deviation (*n* = 3). Statistically significant differences for each protein were assessed using an ordinary one-way ANOVA with Dunnett’s multiple comparison test, compared to control (Ctrl; *, *p* < 0.05, **, *p* < 0.0001). N.D., not detected.

**Figure 3:**
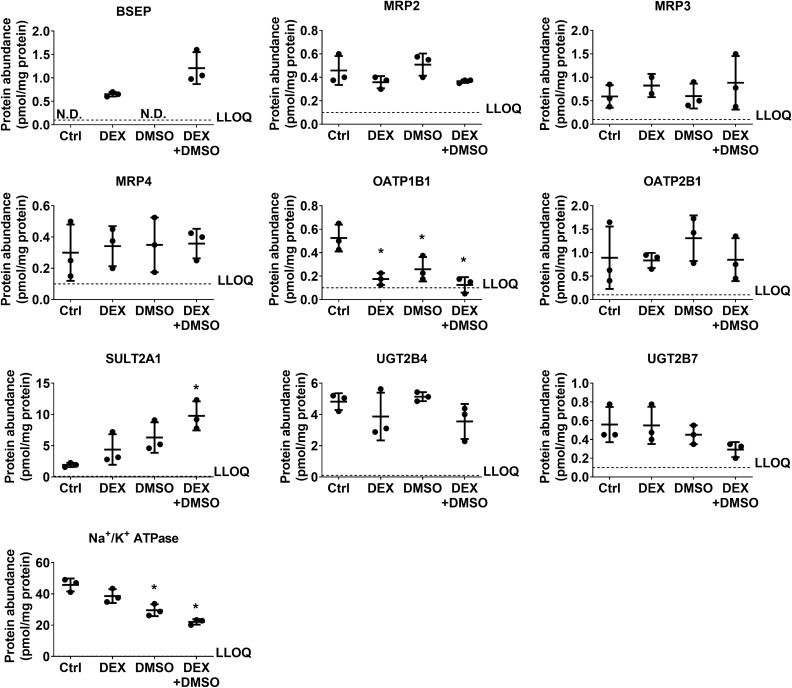
Abundance of bile acid-relevant proteins in differentiated HuH-7 cells with overlay measured by quantitative targeted absolute proteomics (QTAP) analysis. Membrane protein abundance (pmol/mg protein; mean ± standard deviation, *n* = 3) of select bile acid-relevant proteins in 2-week cultured HuH-7 cells treated with 1 µM dexamethasone (DEX) and/or 0.5% DMSO was plotted as scatter plots for individual proteins. Statistically significant differences with respect to control (Ctrl) were calculated for each protein using an ordinary one-way ANOVA and Dunnett’s multiple comparison test (*, *p* < 0.05). N.D., not detected; LLOQ, lower limit of quantitation (0.1 pmol/mg protein).

Protein abundance of bile acid transporters and enzymes was measured using quantitative targeted absolute proteomics (QTAP), with Western blotting serving as an orthogonal approach for confirmation. Proteins that were not detected by proteomics (NTCP, OATP1B3, and OSTα/β) were evaluated using Western blotting. Membrane protein abundance of BSEP was significantly induced in HuH-7 cells cultured with DEX and overlay (Fig. 2a), in agreement with previous findings^13^. DEX addition alone increased BSEP membrane protein by 11-fold compared to the control cells (Ctrl; Fig. 2b). Addition of DMSO to the control and DEX-containing culture medium increased BSEP expression by threefold and 31-fold, respectively, in overlaid cells (Fig. 2b). Similarly in non-overlaid cells, DEX, and DEX + DMSO addition to the culture medium increased BSEP protein expression by fivefold and tenfold, respectively (Supplementary Fig. S1). Consistent with Western blot analysis, QTAP analysis revealed that membrane protein abundance of BSEP increased from 0.65 ± 0.05 pmol/mg protein in overlaid HuH-7 cells cultured with DEX to 1.21 ± 0.34 pmol/mg protein in overlaid cells cultured with DEX + DMSO (Fig. 3).

NTCP protein abundance tended to increase in overlaid HuH-7 cells cultured with DEX or DMSO and was significantly higher when cells were cultured with DEX + DMSO (Fig. 2b). A similar tendency was observed in non-overlaid cells (Supplementary Fig. S1). In overlaid HuH-7 cells, OSTα protein levels decreased by three- to fourfold when cultured with DEX, DMSO or DEX + DMSO compared to control (Fig. 2b). Similarly, OSTα protein levels were reduced by DMSO and DEX + DMSO in HuH-7 cells without overlay (Supplementary Fig. S1). OSTβ protein levels were decreased by fourfold in overlaid cells cultured with DEX (Fig. 2b) while this effect was barely discernable in non-overlaid HuH-7 cells (Supplementary Fig. S1). MRP4 membrane protein abundance was variable or not significantly altered when HuH-7 cells were cultured with DEX + DMSO (Figs. 2, 3, and Supplementary Fig. S1).

Several other proteins involved in bile acid disposition were altered in differentiated HuH-7 cells compared to controls. OATP1B1 and OATP1B3 abundance decreased significantly by up to 4.2-fold (Fig. 3) and 5.5-fold (Fig. 2b and Supplementary Fig. S1), respectively, with DEX, DMSO, and DEX + DMSO addition to HuH-7 cells with and without overlay. Protein abundance of SULT2A1 increased significantly by 5.1-fold with DEX + DMSO (Fig. 3). Interestingly, UGT2B7 abundance showed a decreasing trend with DMSO addition. Na^+^/K^+^ ATPase also was significantly lower with DMSO and DEX + DMSO addition. The abundance of other proteins that play a role in bile acid homeostasis, MRP2, MRP3, OATP2B1 and UGT2B4 was either variable or not statistically different when DEX, DMSO or DEX + DMSO was added to the culture medium (Fig. 3). The proteomic analyses of the membrane protein abundance in overlaid HuH-7 cells indicated that phase I metabolic enzymes were largely increased in cells cultured with DEX ± DMSO (Supplementary Fig. S2). P-gp membrane abundance decreased by 5.5-fold to 0.43 ± 0.26 pmol/mg protein, while CYP3A4 was detected at low levels, approximately 1 pmol/mg protein, with DEX + DMSO addition (Supplementary Fig. S2).

### Major bile acid transporters were properly localized on the apical or basolateral membranes in differentiated HuH-7 cells

**Figure 4:**
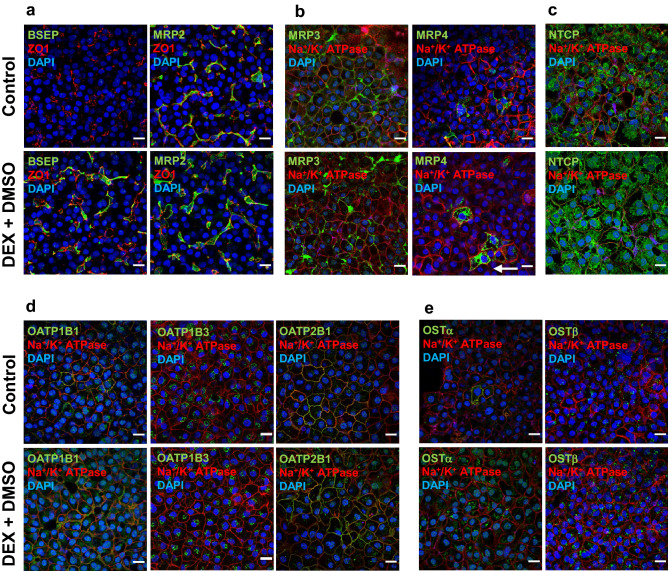
Cellular localization of bile acid transporters in control and differentiated HuH-7 cells with overlay. Immunofluorescence and confocal microscopy were performed to examine transporter localization in control and differentiated [culture medium contained 1 µM dexamethasone (DEX) + 0.5% DMSO] HuH-7 cells with overlay. Immunostaining of zonula occludens 1 (ZO1) and Na^+^/K^+^ ATPase were used as markers for the canalicular and basolateral membranes, respectively. The nucleus was stained with 4′,6-diamidino-2-phenylindole (DAPI). Localization of the following bile acid transport proteins was determined in control and differentiated HuH-7 cells (**a**) bile salt export pump (BSEP) and multidrug resistance-associated protein (MRP) 2, (**b**) MRP3 and MRP4 (white arrow indicates membrane localized protein), (**c**) sodium taurocholate co-transporting polypeptide (NTCP), (**d**) organic anion transporting polypeptide (OATP) 1B1, OATP1B3, and OATP2B1, and (**e**) organic solute transporter (OST) α and OST β. At least three areas were randomly imaged for each sample. All projected images were analyzed using ImageJ software. Scale bar = 20 µm.

As indicated by co-localization with the apical marker ZO1, BSEP was localized predominantly on the canalicular membrane in differentiated HuH-7 cells but was undetectable in overlaid control cells, while MRP2 was found on the canalicular membrane in both culture conditions (Fig. 4a and Supplementary Fig. S3). MRP3 was localized primarily on the basolateral membrane in both the differentiated and control HuH-7 cells with overlay, as indicated by co-localization with the basolateral membrane marker Na^+^/K^+^ ATPase (Fig. 4b and Supplementary Fig. S4). Only a few HuH-7 cells expressed MRP4 regardless of the culture conditions; MRP4 was mainly localized intracellularly, although, some vesicular MRP4 staining also was observed at the basolateral membrane (Fig. 4b and Supplementary Fig. S4). NTCP was mostly intracellularly localized, although, some HuH-7 cells had NTCP on the basolateral membrane in both differentiated HuH-7 and control cells with overlay (Fig. 4c and Supplementary Fig. S5). OATP1B1 was expressed in some HuH-7 cells in both culture conditions and was localized on the basolateral membrane as well as intracellularly around the perinuclear area (Fig. 4d and Supplementary Fig. S5). OATP1B3 was localized intracellularly in most differentiated and control HuH-7 cells (Fig. 4d and Supplementary Fig. S5). OATP2B1 was localized predominantly on the basolateral membrane in both culture conditions in many HuH-7 cells (Fig. 4d and Supplementary Fig. S6). OSTβ localization was primarily intracellular in both culture conditions. Notably, OSTα was on the plasma membrane in very few cells in the control, while in differentiated HuH-7 cells with overlay it was primarily intracellular. (Fig. 4e and Supplementary Fig. S6).

### Differentiation of HuH-7 cells promoted formation of conjugated bile acid species and increased mRNA of bile acid-synthesizing enzymes

**Figure 5:**
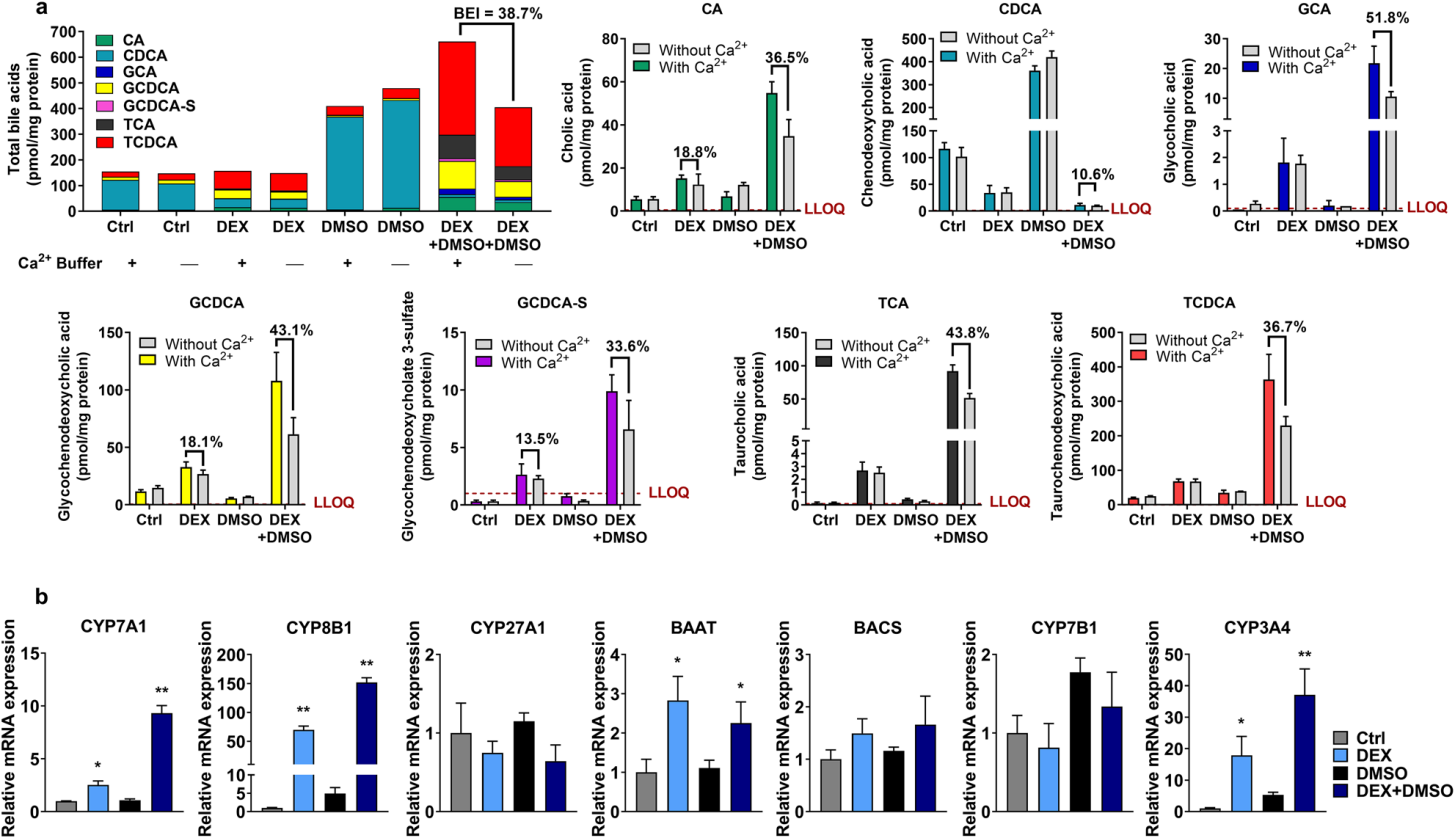
Bile acid profiling and gene expression analysis in HuH-7 cells with overlay. (**a**) Cellular bile acids were measured in overlaid HuH-7 cells cultured for 2 weeks without (Ctrl) and with 1 µM dexamethasone (DEX) and/or 0.5% DMSO using liquid chromatography with tandem mass spectrometry (LC–MS/MS). HuH-7 cells were incubated in standard Hanks' balanced salt solution (HBSS; With Ca^2+^) or Ca^2+^-free HBSS buffer (Without Ca^2+^) for 10 min at 37˚C prior to analysis for determination of “Cells + Bile” and “Cells” content, respectively. Average total bile acid species were plotted based on HuH-7 cell culture conditions. Concentrations of each bile acid species [cholic acid (CA), chenodeoxycholic acid (CDCA), glycocholic acid (GCA), glycochenodeoxycholic acid (GCDCA), GCDCA 3-sulfate (GCDCA-S), taurocholic acid (TCA), and taurochenodeoxycholic acid (TCDCA)] were calculated, normalized to total protein, and plotted as mean ± standard deviation (*n* = 3) based on HuH-7 cell culture conditions. In all cases, the biliary excretion index (BEI) was calculated using Eq. (1), and BEI values (%) were included above the relevant data in the graphs, when measurable. The lower limit of quantitation was 0.1 nM for GCA, TCA and TCDCA, 0.5 nM for GCDCA and CA, and 1 nM for CDCA and GCDCA-S. (**b**) mRNA was measured by RT-qPCR in HuH-7 cells cultured for 2 weeks with overlay, 1 µM DEX and/or 0.5% DMSO. Threshold cycle (C_T_) values of each gene of interest [*cytochrome P450* (*CYP) 7A1*, *CYP8B1*, *CYP27A1, bile acid-CoA: amino acid N-acyltransferase (BAAT), bile acid-CoA synthase (BACS), CYP7B1,* and *CYP3A4*] were normalized to the housekeeping gene β-actin (*ACTB*) and compared with control (Ctrl; set to 1.0). Data are plotted as mean ± standard deviation (n = 3). Statistically significant differences in relative mRNA were determined by an ordinary one-way ANOVA with Dunnett’s multiple comparison test (* *p*-value < 0.05, ** < 0.0001, DEX, DMSO or DEX + DMSO vs. Ctrl).

The endogenous bile acid profile in control and differentiated HuH-7 cells was evaluated using liquid chromatography with tandem mass spectrometry (LC–MS/MS), which revealed a shift in bile acid composition with DEX addition. CDCA was the most abundant bile acid in control and DMSO-supplemented HuH-7 cells (Fig. 5a). With the addition of DEX alone, the formation of CA and conjugated bile acid species, GCA, GCDCA, GCDCA 3-sulfate (GCDCA-S), TCA and TCDCA was promoted, and CDCA was decreased. Compared to control, DMSO addition increased the CDCA content distinctly while all other bile acid species remained relatively unchanged. Addition of DEX + DMSO increased the total bile acid content most remarkably and further increased the synthesis of CA, GCA, GCDCA, GCDCA-S, TCA and TCDCA, compared to DEX or DMSO alone and control HuH-7 cells. GDCDA-S content was increased in differentiated HuH-7 cells, consistent with higher SULT2A1 abundance. Most notably, DEX + DMSO supplementation reduced the CDCA content compared to DMSO alone. Furthermore, using the B-CLEAR methodology, bile acids were quantified after a 10-min exposure to standard (with Ca^2+^) and Ca^2+^-free (without Ca^2+^) buffer, which disrupted tight junctions and enabled measurement of the bile acid content in “Cells + Bile” and “Cells”, respectively. From this data, the total mass of bile acids excreted into bile canaliculi was calculated and the biliary excretion index (BEI) was estimated using Eq. (1). The BEI of endogenous total bile acids (38.7%) and individual bile acid species [CA (36.5%), CDCA (10.6%), GCA (51.8%), GCDCA (43.1%), GCDCA-S (33.6%), TCA (43.8), and TCDCA (36.7%)] (Fig. 5a) was consistent with functional excretion of bile acids from differentiated HuH-7 cells into the canalicular-like compartment by BSEP.

To further understand the decrease in CDCA in differentiated HuH-7 cells, the mRNA of bile acid synthesizing enzymes was measured in overlaid HuH-7 cells. Relative to control HuH-7 cells, CYP7A1 mRNA was increased 2.5- and 9.3-fold in cells cultured with DEX and DEX + DMSO, respectively (Fig. 5b). Similarly, the mRNA for CYP8B1, which drives higher CA and lower CDCA synthesis^24^, was induced 70- and 152-fold by DEX and DEX + DMSO, respectively. BAAT enzyme mRNA showed a modest but significant two- to threefold increase with DEX and DEX + DMSO. Additionally, the mRNA for CYP3A4, a major drug-metabolizing enzyme, was upregulated 18-fold by DEX and 37-fold by DEX + DMSO. While DEX or DMSO alone increased CYP7A1, CYP8B1 and CYP3A4 mRNA in overlaid HuH-7 cells, the effect of DEX + DMSO was synergistic on all three CYP mRNAs. CYP27A1, BACS, and CYP7B1 mRNA were not significantly different with DEX ± DMSO addition.

### [^3^H]-TCA uptake and biliary excretion were mediated by NTCP and BSEP, respectively, in differentiated HuH-7 cells

**Figure 6:**
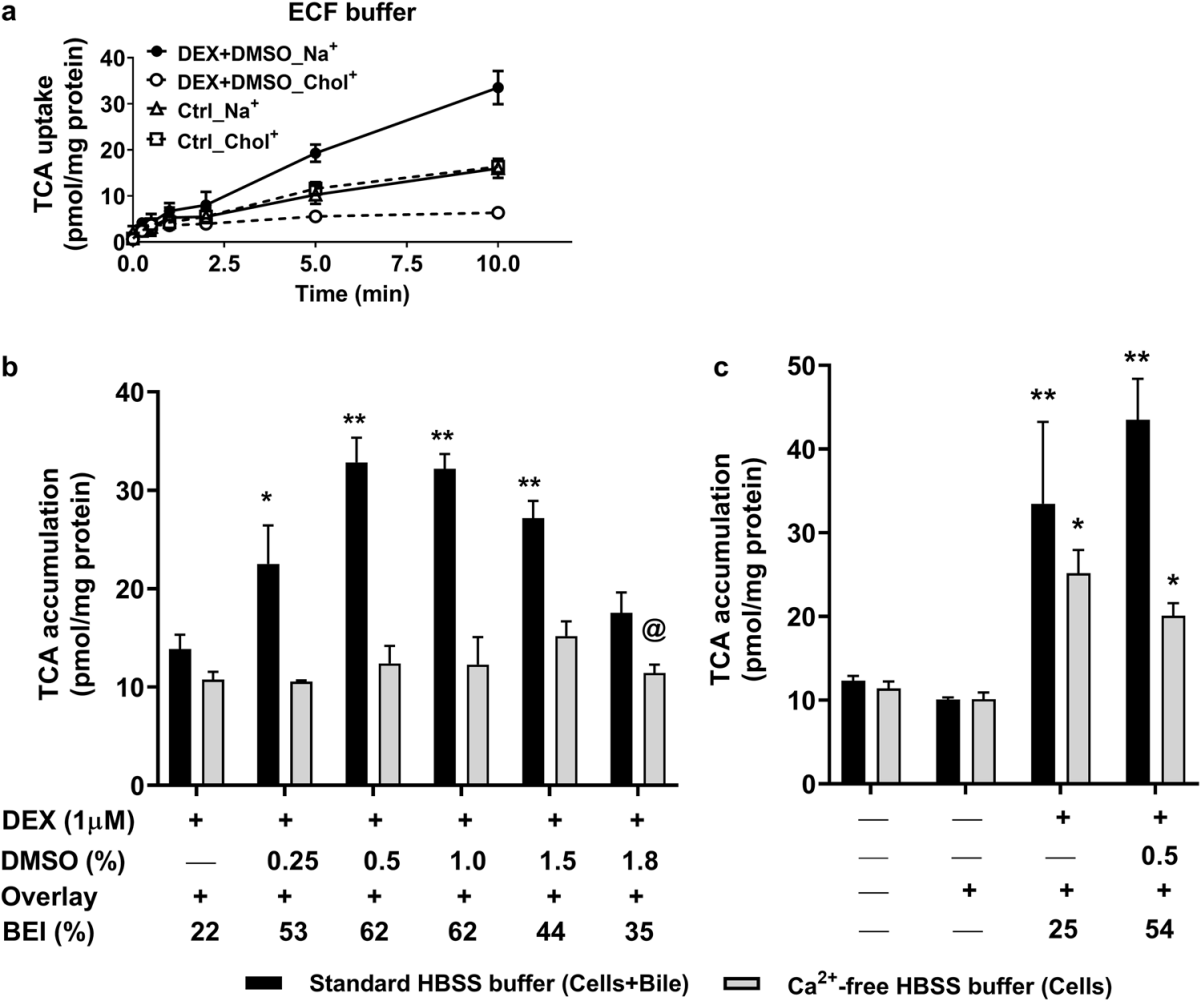
Effect of dexamethasone (DEX) and DMSO on bile acid uptake and biliary excretion index (BEI) in differentiated HuH-7 cells. (**a**) Uptake of 4 µM taurocholic acid ([^3^H]-TCA; 400 nCi/mL) was measured in control (Ctrl) and differentiated (1 µM DEX + 0.5% DMSO) HuH-7 cells without overlay using extracellular fluid (ECF; N = 2, in triplicate) buffer over 10 min. (**b**) Accumulation and BEI of 2 µM [^3^H]-TCA (200 nCi/mL) were measured in HuH-7 cells with overlay in standard or Ca^2+^-free Hanks balanced salt solution buffer (HBSS) at various DMSO concentrations, and (**c**) in the presence and absence of DEX, overlay and 0.5% DMSO. Data were plotted as mean ± standard deviation (*n* = 3) and BEI was calculated using Eq. (1). Statistically significant differences were measured using an ordinary two-way ANOVA with Dunnett’s multiple comparisons test [*, *p* < 0.05, **, *p* < 0.0001, (**b**) cells cultured with overlay and with DMSO vs. without DMSO; (**c**) control cells cultured without vs with overlay and cells cultured with overlay with DEX ± DMSO]. ^@^Cell detachment observed.

Non-overlaid differentiated HuH-7 cells were used to distinguish between Na^+^-dependent NTCP-mediated and Na^+^-independent OATP-mediated bile acid uptake. Using [^3^H]-TCA as a probe bile acid substrate, NTCP-mediated uptake was measured as the difference in [^3^H]-TCA uptake between the Na^+^-containing extracellular fluid (ECF) buffer and the Na^+^-free (Chol^+^) ECF buffer. [^3^H]-TCA uptake at 10 min in differentiated HuH-7 cells exposed to Na^+^-containing ECF buffer was 33.5 pmol/mg protein and in Chol^+^ ECF buffer (Na^+^-independent uptake) was 6.3 pmol/mg protein (Fig. 6a). Therefore, Na^+^-dependent or NTCP-mediated bile acid uptake (27.2 pmol/mg protein) accounted for 81% of total [^3^H]-TCA uptake. Additionally, [^3^H]-TCA uptake at 10 min was 2.5-fold lower in differentiated HuH-7 cells in Chol^+^ buffer compared to control cells, which is consistent with lower OATP1B1 and OATP1B3 protein in DEX + DMSO-supplemented HuH-7 cells. [^3^H]-TCA uptake was similar in control cells exposed to Na^+^- and Chol^+^-containing ECF buffers, indicating an absence of Na^+^-dependent NTCP-mediated uptake.

Since TCA is a prototypical substrate for BSEP^33^, the BEI of exogenously administered [^3^H]-TCA was determined in differentiated HuH-7 cells cultured in the presence and absence of DEX, overlay, and at different DMSO concentrations (Fig. 6b and c). Accumulation of [^3^H]-TCA in “Cells + Bile” was significantly higher when cells were cultured in the presence of DEX with all DMSO concentrations except 1.8% DMSO with DEX, where cell detachment was observed (Fig. 6b), and highest with the addition of 0.5% DMSO with DEX. [^3^H]-TCA accumulation in “Cells” did not vary significantly when cells were cultured in the presence of DEX with increasing DMSO concentrations. The highest BEI was observed in cells cultured with 0.5% and 1% DMSO with DEX. Based on all these findings, 0.5% DMSO was selected for subsequent experiments. HuH-7 cells cultured without DEX did not exhibit any BEI (Fig. 6c) similar to previous findings in 4-week cultured HuH-7 cells^13^. HuH-7 cells cultured with DEX, DEX + DMSO and overlay showed increased [^3^H]-TCA accumulation in “Cells + Bile” and in “Cells” compared to control cells with and without overlay. While addition of DEX alone to the culture medium enhanced BSEP-mediated BEI to 25%, addition of 1 µM DEX + 0.5% DMSO increased BEI to 54% in differentiated HuH-7 cells (Fig. 6c). The apparent in vitro uptake clearance (CL_uptake,app_) and biliary clearance (CL_biliary,app_) values for [^3^H]-TCA were calculated using Eqs. 2 and 3, respectively (Fig. 6b and c). The CL_uptake,app_ and CL_biliary,app_ increased when HuH-7 cells were cultured with DEX + DMSO (mean values of 1.9 and 0.9 µl/min/mg protein, respectively; Table 1) compared to 1.2 and 0.3 µl/min/mg protein (DEX alone). The experiments in Figs. 1, 2, 3, 4, 5, 6 were conducted with cells at passages 12–28 because [^3^H]-TCA accumulation increased significantly at a higher passage number (Supplementary Fig. S7).

**Table 1:** Summary of biliary excretion index (BEI), apparent in vitro uptake clearance (CL_uptake,app_) and biliary clearance (CL_biliary,app_) of radiolabeled taurocholate ([^3^H]-TCA) in differentiated HuH-7 cells compared with previously published data in HepaRG cells and human hepatocytes^13^. Differentiated HuH-7 cell data are from Figs. 6b, c, and 7.

	Differentiated HuH-7 cells	HepaRG	Human hepatocytes
BEI (%)	30–62	27–39	30–75
CL_uptake,app_ (µl/min/mg protein)	1.6–2.2	2.7–13.8	2.7–20
CL_biliary,app_ (µl/min/mg protein)	0.6–1.2	1–4	0.8–25

### Differentiated HuH-7 cells as an in vitro model to examine cholestatic hepatotoxicity

**Figure 7:**
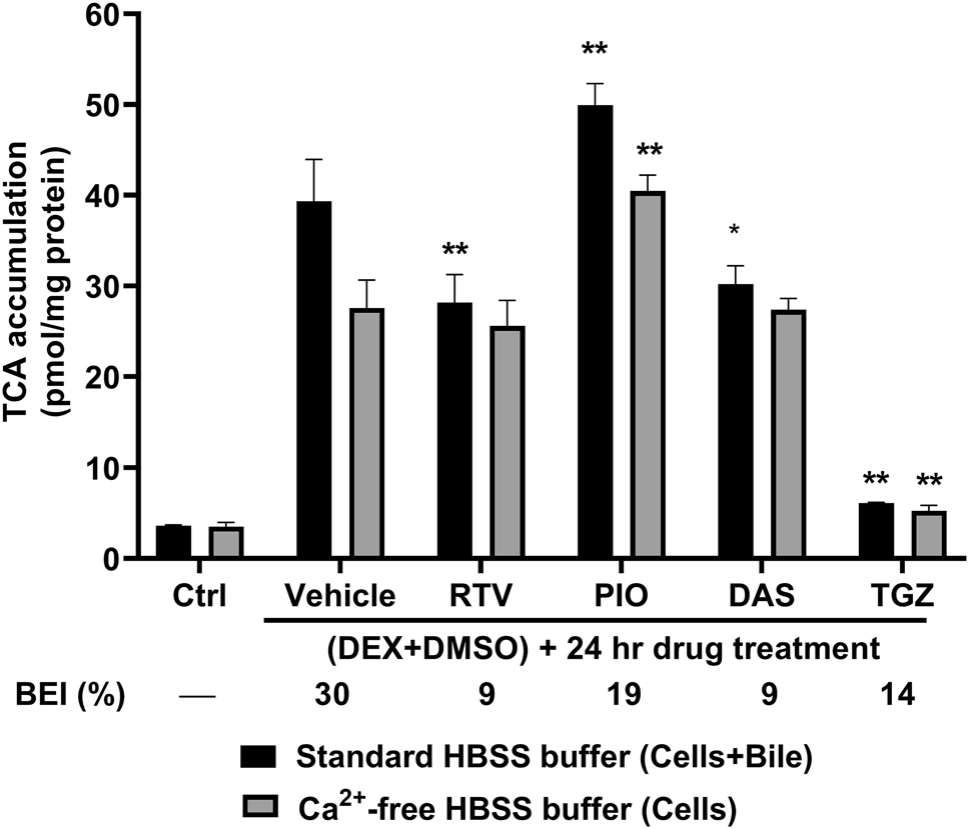
Effect of cholestatic drugs on the accumulation and biliary excretion index (BEI) of taurocholate (TCA) in control and differentiated HuH-7 cells with overlay. Accumulation and BEI of 2 µM [^3^H]-TCA (200 nCi/mL) were measured in control (untreated; Ctrl) and differentiated (1 µM DEX + 0.5% DMSO) HuH-7 cells with overlay in standard or Ca^2+^-free HBSS buffer after a 24-h incubation with cholestatic drugs. On day 13 of culture, differentiated HuH-7 cells were exposed to 0.1% DMSO control (vehicle), ritonavir (RTV; 25 µM), pioglitazone (PIO; 100 µM), dasatinib (DAS; 20 µM), or troglitazone (TGZ; 75 µM) for 24 h. Data were plotted as mean ± standard deviation (*n* = 3) and biliary excretion index (BEI, %) was calculated using Eq. (1). Statistically significant differences were measured using an ordinary two-way ANOVA with Dunnett’s multiple comparisons test (*, *p* < 0.05, **, *p* < 0.0001, vehicle control vs. drug treatment).

Changes in bile acid transporter function were assessed using the B-CLEAR assay in differentiated HuH-7 cells pretreated with prototypical cholestatic drugs for 24 h (Fig. 7). [^3^H]-TCA accumulation in “Cells + Bile” (Standard Buffer) was significantly decreased by ritonavir (25 µM), dasatinib (20 µM), and troglitazone (75 µM), but significantly increased by pioglitazone (100 µM) compared to vehicle control (0.1% DMSO). Troglitazone treatment decreased [^3^H]-TCA accumulation while pioglitazone increased [^3^H]-TCA accumulation in “Cells” (Ca^2+^-free Buffer). Compared to vehicle control, the BEI of [^3^H]-TCA in differentiated HuH-7 cells was lower for all cholestatic drugs after a 24-h incubation.

## Discussion

In this report, HuH-7 cells were differentiated in two weeks using DEX and DMSO culture supplements and Matrigel overlay. Novel findings in 2-week cultured differentiated HuH-7 cells include: (1) improved membrane protein abundance and function of BSEP, (2) increased NTCP membrane protein and function, (3), decreased OATP1B1 and OATP1B3 protein and function, (4) increased SULT2A1 protein and higher GCDCA-S content, (4) proper membrane localization of hepatic bile acid transporters, (5) decreased OSTα/β that is indicative of restored hepatic function, (6) increased CA and lower CDCA content, consistent with increased CYP8B1 mRNA, (7) increased abundance of glycine- and taurine-conjugated bile acids, consistent with higher BAAT mRNA, and (8) the CL_uptake,app_, CL_biliary,app_, and BEI of [^3^H]-TCA were comparable with sandwich-cultured human hepatocytes (SCHH). Additionally, the differentiated HuH-7 cell model was sensitive to cholestatic drugs and displayed altered [^3^H]-TCA disposition that may contribute to hepatotoxicity.

Glucocorticoids such as DEX are commonly added to primary hepatocyte cultures to promote CYP activity, cytoskeletal re-arrangement, enhance gap junction expression and function and improve formation of bile canalicular networks^34^. DEX is a glucocorticoid receptor agonist, which in turn induces the expression of CAR and PXR in human hepatocytes^15,16^. Previous publications indicated that DMSO (1%) addition to HuH-7 cell cultures for 20 days resulted in a differentiated, growth-arrested state with CAR induction, increased CYP and UGT mRNA and activity^22^. The same effect was observed in the present study for CYP 8B1 and CYP3A4 mRNA in HuH-7 cells cultured for 2 weeks with 0.5% DMSO alone. In previously published studies, DMSO promoted differentiation of stem cells and rat hepatocytes, and increased hepatic transcriptional factors such as hepatocyte nuclear factor-4α^35–38^. DMSO, as low as 0.1% (v/v) over two weeks, induced drastic cellular and epigenetic changes in hepatic tissues, where genes associated with metabolism and vesicle-mediated transport were most affected^39^. DMSO (2%) and hydrocortisone were also required for the proper differentiation of HepaRG cells over two weeks of culture^40^. However, the molecular targets of DEX and DMSO that lead to synergistic changes in key bile acid-relevant enzymes and transporters were not identified in HepaRG cells or differentiated HuH-7 cells. Extracellular matrices such as Matrigel are used for hepatocyte culture to improve cell polarity, hepatocyte-like morphology, and canalicular formation^13,41^.

Consistent with previous findings in 4-week overlaid HuH-7 cultures^13^, DEX alone increased BSEP expression and function in overlaid HuH-7 cells compared with control, as measured by B-CLEAR; the BEI of [^3^H]-TCA was 25% in this study. In 2-week differentiated HuH-7 cells, addition of DEX + DMSO to the culture medium further increased BSEP expression and function compared to DEX alone, with retained apical localization. In differentiated HuH-7 cells, membrane protein abundance of BSEP (1.21 ± 0.34 pmol/mg protein), as measured by proteomics, was higher than that in suspended, plated or SCHH (0.57 ± 0.20 pmol/mg protein)^42^. Compared to control, MRP2 expression and localization were unchanged with DEX alone as reported previously^13^. A similar effect was observed with DEX + DMSO addition. MRP2 membrane protein abundance in differentiated HuH-7 cells (0.37 ± 0.01 pmol/mg protein), was within the range reported for human hepatocytes (0.76 ± 0.52 pmol/mg protein)^42^. Membrane-bound P-gp protein showed a decreasing trend in 4-week overlaid HuH-7 cells cultured with DEX^13^, while in 2-week differentiated HuH-7 cells, P-gp membrane abundance decreased significantly but maintained a level similar to human hepatocytes (0.25 ± 0.24 pmol/mg protein)^42^.

This is the first study to report evidence of functional NTCP induced in HuH-7 cells by differentiation: membrane abundance of NTCP was significantly increased and NTCP-mediated (Na^+^-dependent) [^3^H]-TCA uptake accounted for 81% of total TCA uptake in differentiated HuH-7 cells, similar to human hepatocytes^43,44^. This differentiated HuH-7 cell model with expression of functional NTCP may prove valuable in the development of drugs against hepatitis B, given that NTCP is the main entry receptor for hepatitis B virus^45^. Also shown here for the first time, OATP1B1 and OATP1B3 membrane protein and function were decreased similarly in 2-week cultured HuH-7 cells with DEX, DMSO or DEX + DMSO addition. OATP1B1 (0.13 ± 0.07 pmol/mg protein) and OATP2B1 (0.85 ± 0.46 pmol/mg protein) were present on the basolateral membrane in differentiated HuH-7 cells at lower and similar membrane abundance compared to human hepatocytes (1.20 ± 0.69 and 0.52 ± 0.13 pmol/mg protein, respectively)^42^. MRP3 membrane abundance in differentiated HuH-7 cells (0.88 ± 0.57 pmol/mg protein) was variable but within the range of human hepatocytes (0.39 ± 0.25 pmol/mg protein), while MRP4 is typically of low abundance and undetected in human hepatocytes^42,46^. The signature peptides used for QTAP by Kumar et al. were the same as this study for BSEP, MRP2 and OATP1B1 but different for P-gp and OATP2B1^42^. Since OSTα and OSTβ are upregulated in cholestatic diseases and are undetected or of low abundance in normal liver^47,48^, the decline in both OSTα and OSTβ abundance with DEX + DMSO addition suggests that differentiation restored normal hepatic function in HuH-7 cells. In summary, key bile acid transporters generally were expressed at or near levels reported in SCHH. These novel findings suggest that HuH-7 cells differentiated with DEX + DMSO no longer function as cholestatic cells^12^.

Additional evidence for restored hepatic function was provided by the remarkable shift in bile acid composition and decreased CDCA content with differentiation. This is significant because higher cellular CDCA has been associated with cholestasis^49^. Lower CDCA was expected because CYP8B1 mRNA increased with DEX ± DMSO supplementation, which should increase CA synthesis and decrease CDCA synthesis based on the classical bile acid synthesis pathway in humans^24^. Consistent with these findings, DEX-mediated upregulation of CYP8B1 mRNA and increased CA synthesis were reported previously in primary human hepatocytes^50^. Higher bile acid content in differentiated HuH-7 cells was also driven by increased CYP7A1 activity, which is the rate-limiting step in bile acid synthesis^51^. Overall, CYP enzyme mRNA and function are well correlated^52^ and CYP7A1 overexpression was shown to increase activity and bile acid synthesis^53^. Increased bile acid conjugation with taurine and glycine was consistent with increased BAAT mRNA in HuH-7 cells cultured with medium containing DEX or DEX + DMSO. Furthermore, the increased GCDCA-S content in differentiated HuH-7 cells was consistent with increased SULT2A1 protein. Overall, phase 1 metabolic enzymes increased with differentiation, similar to previous reports^10^.

The BEI of total and individual bile acid species was quantified in differentiated HuH-7 cells. The CL_uptake,app_ and BEI of [^3^H]-TCA in differentiated HuH-7 cells were comparable to that in SCHH and HepaRG cells (Table 1)^13,54,55^. The CL_biliary,app_ of [^3^H]-TCA was higher in 2-week differentiated HuH-7 cells than in 4-week cultured HuH-7 cells with DEX alone, but within the reported range for SCHH and HepaRG cells^13^. The BEI of endogenous GCA, GCDCA, TCA, TCDCA and total bile acids in differentiated HuH-7 cells was similar or higher than the values reported in SCHH^56^. The BEI of endogenous CA, CDCA and GCDCA-S as measured in differentiated HuH-7 cells has not been reported in human hepatocytes. In comparison to SCHH, total bile acids were 8-to 14-fold lower in differentiated HuH-7 cells^57^.

To investigate the potential application of this in vitro model to predict cholestatic toxicity, differentiated HuH-7 cells were utilized to measure a change in BEI of [^3^H]-TCA when exposed to various hepatotoxic drugs for 24 h. Ritonavir, pioglitazone, dasatinib, and troglitazone were chosen as prototypical hepatotoxic compounds that inhibited BSEP, MRP3, and/or MRP4 in membrane vesicle studies and altered bile acid disposition in SCHH^58^. Ritonavir, dasatinib and troglitazone were reported previously to cause bile acid-dependent toxicity in SCHH at concentrations similar to this study^57,59^. Ritonavir decreased [^3^H]-TCA accumulation in “Cells + Bile” and BEI in SCHH and 4-week overlaid HuH-7 cells with DEX addition, similar to differentiated HuH-7 cells, due to inhibition of NTCP- and OATP-mediated uptake, and BSEP-mediated biliary excretion^59,60^. In 2-week and 4-week differentiated HuH-7 cells, pioglitazone treatment increased [^3^H]-TCA accumulation in “Cells + Bile” and “Cells”, while lowering BEI, consistent with BSEP inhibition^59,61^. Although pioglitazone inhibits OATP1B1-, OATP1B3- and NTCP-mediated bile acid uptake^62^, higher [^3^H]-TCA accumulation in “Cells + Bile” and “Cells” may occur due to BSEP and MRP4 inhibition^58^. Compared to vehicle control, dasatinib lowered [^3^H]-TCA BEI in differentiated HuH-7 cells similar to SCHH, consistent with BSEP inhibition^57^. Troglitazone response also was consistent in differentiated HuH-7 cells and SCHH; here, [^3^H]-TCA accumulation in “Cells + Bile” and “Cells” and also BEI were decreased compared to vehicle control^59^.

In conclusion, these results demonstrate that addition of DEX, DMSO, and Matrigel restored bile acid metabolism and transport in HuH-7 cells after two weeks of culture. BSEP inhibition in membrane vesicles and SCHH by these cholestatic hepatotoxic drugs was recapitulated in differentiated HuH-7 cells. The differentiated HuH-7 cell model with restored hepatic function provides a novel, readily available cellular system for investigation of transporter regulation mechanisms and could be used in high-throughput screening of compounds for cholestatic hepatotoxicity.

## Materials and methods

### Chemicals and reagents

DEX (catalog 11,015) was purchased from Cayman Chemicals (Ann Arbor, MI). [^3^H]-TCA (#NET322250UC, > 97% radiochemical purity) was obtained from PerkinElmer Inc. (Boston, MA). DMSO, HPLC grade acetonitrile, methanol, water, formic acid, and other reagents were from Sigma-Aldrich (St. Louis, MO) or Fisher Scientific (Pittsburg, PA).

### HuH-7 cell culture

The human hepatoma HuH-7 cell line (JCRB0403) was purchased from Sekisui Xenotech (Kansas City, KS). Cells were cultured in maintenance medium [Dulbecco’s modified Eagle’s medium (DMEM, #11995-065, Thermo Fisher Scientific), 10% fetal bovine serum (FBS; #F2442, Sigma-Aldrich), 100 U/ml penicillin, and 100 μg/ml streptomycin (#10378–016, Thermo Fisher Scientific)]^13^. The identity of the cell line was verified by amplification of 17 short tandem repeats by the UNC Vironomics Core. Extended culture of HuH-7 cells was modified from previously described methods^13^. On day 0, HuH-7 cells were seeded at 0.3–0.4 million cells per well in 24-well plates with a tissue-culture treated surface (#353,226, Corning, Durham, NC). On day 2, the maintenance medium was supplemented with and without 1 μM DEX ± DMSO (v/v; 0.25–1.8% and optimized to 0.5%). On day 7, the cultures were overlaid with 0.25 mg/mL Matrigel Basement Membrane Matrix (#354234, lot 0020005 or 0062014, Corning) in ice-cold maintenance medium with and without 1 μM DEX and/or DMSO (v/v; 0.25 – 1.8% and optimized to 0.5%). Cells were maintained for two weeks with medium renewed every 2–3 days. On day 14, terminal assays were conducted. All experiments were performed between passage numbers 12–28 (Supplementary Fig. S7).

### Gene expression studies using RT-qPCR

Overlaid HuH-7 cells were cultured in the absence and presence of 1 µM DEX and 0.5% DMSO (v/v). RNA was extracted from cells using the TRI Reagent according to the manufacturer’s protocol. The concentration and purity of isolated RNA was measured using a NanoDrop spectrophotometer. Reverse transcription of the RNA (2 µg) to cDNA was performed using the Applied Biosystems High-Capacity cDNA Reverse Transcription Kit. CYP7A1, CYP8B1, CYP27A1, BAAT, BACS, CYP7B1, and CYP3A4 mRNAs were quantified using RT-qPCR for each sample in triplicate with the QuantStudio 6 Flex System. The analyzed genes and the gene-specific TaqMan assays used for RT-qPCR are listed in Supplementary Table S1. Gene expression was calculated using the ∆∆Ct method, where β-actin was used as the housekeeping gene.

### Membrane protein extraction and Western blot analysis

On day 14, the cells were harvested to isolate the membrane and cytosolic fractions from each sample (*n* = 3) using the ProteoExtract Native Membrane Protein Extraction Kit (#444810, Millipore Sigma-Aldrich), following the manufacturer’s instructions. Total protein in the cytosolic and membrane fraction was determined using the Pierce™ BCA Protein Assay Kit (#23225, Thermo Fisher Scientific). Membrane fractions isolated from each sample were used for Western blotting and nano-ultra-high-performance liquid chromatography/tandem mass spectrometry (nanoLC-MS/MS).

Western blotting of membrane proteins (25–35 µg) was conducted as described previously (see Supplementary Methods and Supplementary Table S2 for details). Chemiluminescent signal was detected using SuperSignal West Femto Maximum Sensitivity Substrate (#34096, Thermo Fisher Scientific) and Molecular Imager VersaDoc imaging system (BioRad, Hercules, CA).

### NanoLC-MS/MS-based quantitative targeted absolute proteomic (QTAP) analysis

Membrane protein from each sample (20 µg, n = 3) of 2-week cultured HuH-7 cells was mixed with stable isotope- labeled (SIL) proteotypic tryptic peptides, digested using trypsin, and recovered using solid phase extraction as described previously^46^. Proteomic analysis was performed using a validated nanoLC-MS/MS method^46,63^. The SIL peptides used to report protein abundance and the multiple reaction monitoring (MRM; labeled internal standard, unlabeled analyte) acquired for each peptide are shown in Supplementary Table S3, and were validated previously^46,52,63^. Equality of MRM response between the SIL and unlabeled peptides was assumed, and MultiQuant 2.0.2 software (SCIEX, Framingham, MA) was used for calculation of peak area ratios of unlabeled (endogenous) peptides to SIL peptides unique to each protein. The peak area ratios from two MRM transitions were averaged to calculate protein abundance, based on 1 pmol of SIL added during sample preparation.

### Immunofluorescence and imaging

HuH-7 cells were cultured in glass-bottom dishes with 1 µM DEX and 0.5% DMSO (v/v) in the presence of overlay. To highlight canalicular structures using 5(6)-carboxy-2′,7′-dichlorofluorescein (CDF), cells were washed twice with warm standard Hanks' balanced salt solution (HBSS), followed by an incubation with 2 µM CDF diacetate (CDFDA) for 20 min at 37 °C. Subsequently, cells were washed twice with standard HBSS, and imaging was performed using a Nikon Eclipse TS100 inverted microscope.

HuH-7 cells were cultured in glass-bottom dishes in the absence and presence of 1 µM DEX and 0.5% DMSO (v/v) with overlay for immunofluorescence using methods described in a previous study (see Supplementary Methods and Supplementary Table S2 for details)^64^. Z-stack confocal images were taken using a Zeiss 880 confocal laser scanning microscope with a Plan-Neofluar 40x/1.3 oil WD0.21 objective.

### Assessment of transporter function in HuH-7 cells

To determine the contribution of NTCP to bile acid uptake, the cellular uptake of the probe bile acid substrate [^3^H]-TCA was measured in control and differentiated (1 µM DEX + 0.5% DMSO) HuH-7 cells cultured for 2 weeks without overlay (*n* = 3 per group). On day 14, cells were washed twice with warm extracellular fluid (ECF) buffer with sodium (Na^+^) or choline (Chol^+^), as published previously^12,47^. Subsequently, cells were incubated with 4 μM [^3^H]-TCA (400 nCi/ml) in each buffer for 0.25, 0.5, 1, 2, 5, and 10 min, prior to two washes with cold ECF Na^+^ or ECF Chol^+^ buffers.

The accumulation of bile acids in “Cells + Bile” and ”Cells” was measured using B-CLEAR technology^34,65^ in control and differentiated HuH-7 cells with overlay at various DMSO concentrations and passage numbers (15, 27 and 47). For drug-mediated inhibition of [^3^H]-TCA uptake and excretion, on day 13 of culture, differentiated HuH-7 cells with overlay were exposed for 24 h to various cholestatic drugs such as ritonavir (25 µM), pioglitazone (100 µM), dasatinib (20 µM), troglitazone (75 µM), and 0.1% DMSO control. Cells were washed twice in standard HBSS or Ca^2+^-free HBSS containing 1 mM EGTA followed by a 10-min pre-incubation in standard or Ca^2+^-free HBSS buffer at 37 °C. The standard HBSS buffer containing Ca^2+^ and Mg^2+^ ions represents “Cells + Bile”, whereas the Ca^2+^-free HBSS disrupts tight junctions, and therefore, represents “Cells”^66^. Subsequently, cells were treated with 2 μM [^3^H]-TCA (200 nCi/ml) in standard HBSS for 10 min at 37 °C and washed three times in ice-cold standard HBSS. All plates were frozen at -20 °C until processed further by lysis using 400 μl of 0.5% Triton X-100 and 0.005% Antifoam-A PBS. Radioactivity of cell lysates was measured using Bio-Safe II counting cocktail (Research Products International Corp., Mt Prospect, IL) and a Tri-Carb 3100TR liquid scintillation analyzer (PerkinElmer Inc.). TCA accumulation was normalized to total protein content per well, which was determined using Pierce BCA Protein Assay Kit following the manufacturer’s instructions. The biliary excretion index (BEI), which represents the percentage of total mass accumulated that is excreted into bile, was calculated using Eq. (1). The apparent in vitro uptake clearance (CL_uptake,app_) and biliary clearance (CL_biliary,app_) values were calculated using Eqs. (2) and (3)^34,66^.

1 $$ {\text{BEI}} \left( \% \right) = \frac{{{\text{Accumulation}}_{{\left( {{\text{Cells}} + {\text{Bile}}} \right)}} - {\text{Accumulation}} _{{({\text{Cells}})}} }}{{{\text{Accumulation}} _{{({\text{Cells}} + {\text{Bile}})}} }} \times 100 $$

2 $$ {\text{CL}}_{{{\text{uptake}},\,\, {\text{app}}}} = \frac{{{\text{Accumulation}}_{{({\text{Cells}} + {\text{Bile)}}}} }}{{{\text{Incubation}} {\text{Time}} \times {\text{Concentration}}_{{{\text{media}}}} }} $$

3 $$ {\text{CL}}_{{{\text{biliary}},\,\,{\text{ app}}}} = \frac{{{\text{Accumulation}}_{{({\text{Cells}} + {\text{Bile}})}} - {\text{Accumulation}}_{{({\text{Cells}})}} }}{{{\text{Incubation}} {\text{Time}} \times {\text{Concentration}}_{{{\text{media}}}} }}. $$

### Mass spectrometry-based bile acid profiling

Sample preparation and bile acid quantitation methodology were adapted from previously described methods^67,68^. Overlaid HuH-7 cells were cultured without and with 1 µM DEX and/or 0.5% DMSO (v/v). Cells were washed twice in standard HBSS or Ca^2+^-free HBSS containing 1 mM EGTA followed by a 10-min incubation in standard or Ca^2+^-free HBSS buffer at 37 °C. Cells were washed once with ice-cold standard HBSS buffer and frozen at -80 °C prior to analysis. Abbreviations and sources of bile acid species are shown in Supplementary Table S4. Sample preparation and chromatographic separation were achieved using previously published methods^57^. Calibration curves were performed in HuH-7 cell lysate and the data were acquired with MRM, and the collision energy for each bile acid species is listed in Supplementary Table S4.

### Data analysis

Data were analyzed as described in the figure legends to determine statistically significant differences using GraphPad Prism 7.03.

## Supplementary Information

Supplementary Information 1.

Supplementary Information 2.

## Data Availability

The mass spectrometry proteomics data have been deposited to the ProteomeXchange Consortium via the PRIDE partner repository with the dataset identifier PXD031731. All data generated or analyzed during this study are included in this published article or are available upon request.
