# Supplementary material for: A novel differentiated HuH-7 cell model to examine bile acid metabolism, transport and cholestatic hepatotoxicity
Source: Sci Rep. 2022 Aug 22;12:14333. doi: 10.1038/s41598-022-18174-z (PMC9395349; doi:10.1038/s41598-022-18174-z)

Figure 2a

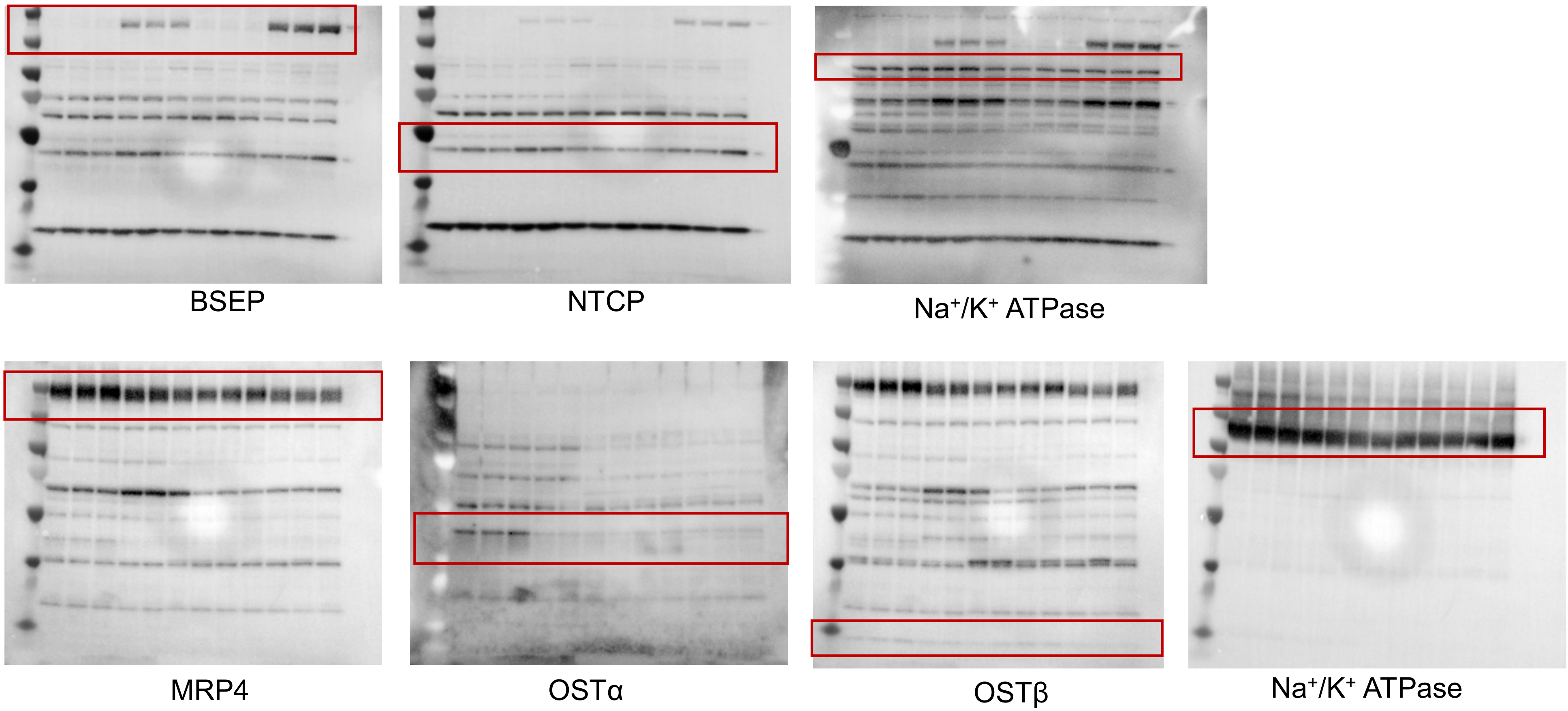

Supplementary Figure S1

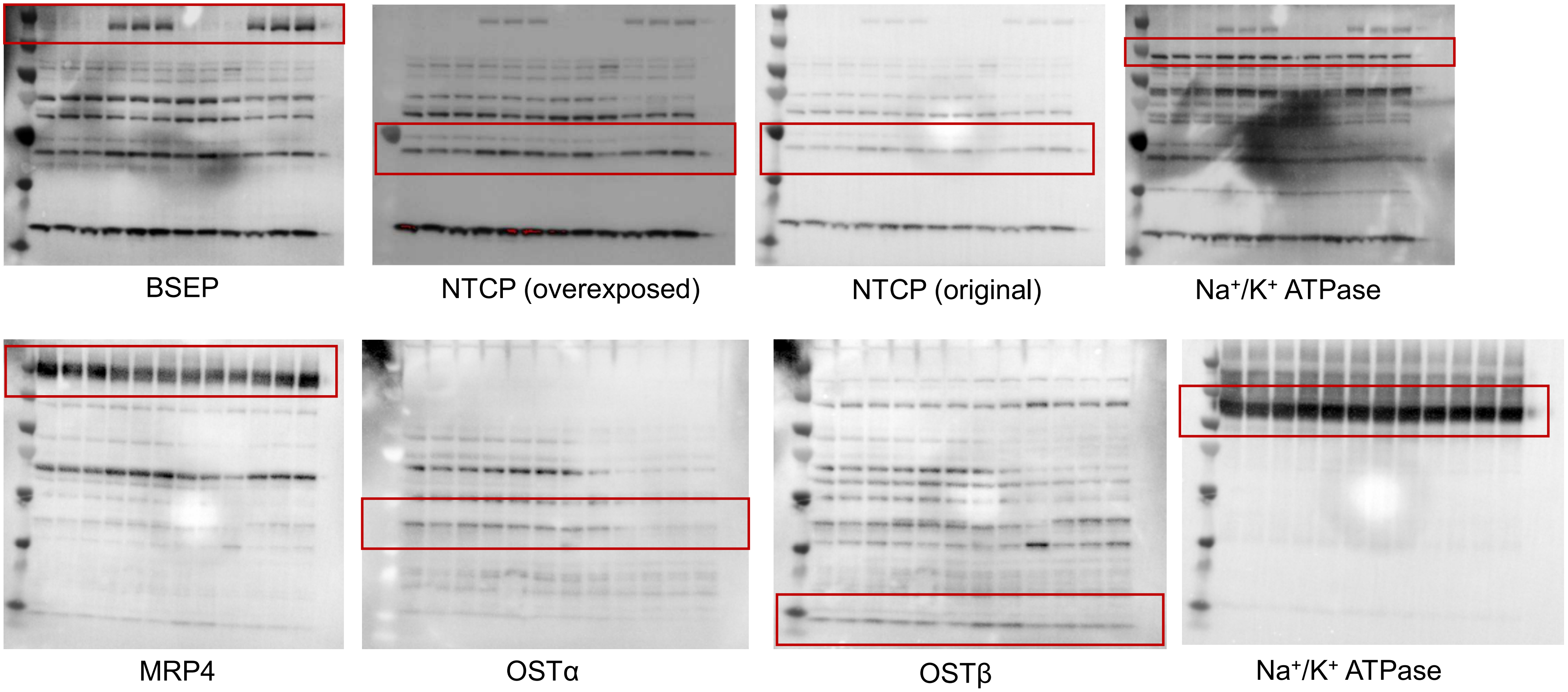

Figure 2a

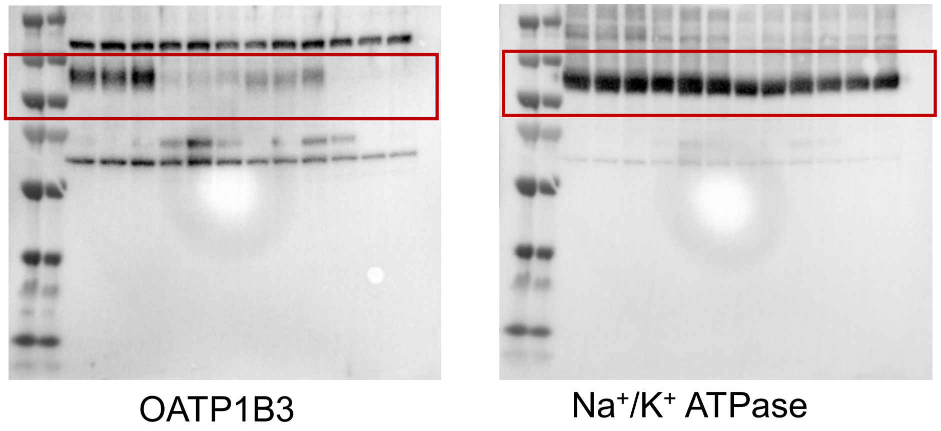

Supplementary Figure S1

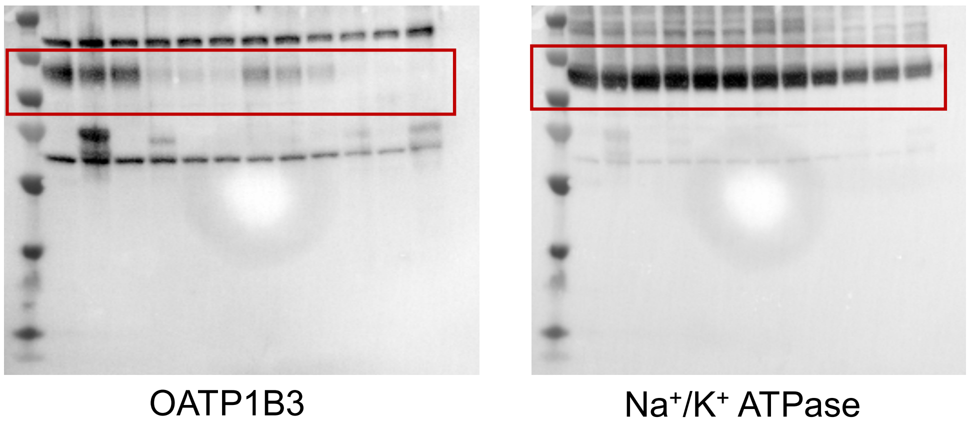

Supplement: Supplementary file 1 — Supplementary Information 1. [file 41598_2022_18174_MOESM1_ESM.pdf]
