# Supplementary material for: A novel differentiated HuH-7 cell model to examine bile acid metabolism, transport and cholestatic hepatotoxicity
Source: Sci Rep. 2022 Aug 22;12:14333. doi: 10.1038/s41598-022-18174-z (PMC9395349; doi:10.1038/s41598-022-18174-z)
Supplement: Supplementary file 2 — Supplementary Information 2. [file 41598_2022_18174_MOESM2_ESM.pdf]

Supplementary Information

A Novel Differentiated HuH-7 Cell Model to Examine Bile Acid Metabolism, Transport and Cholestatic Hepatotoxicity

Chitra Saran[1,2], Dong Fu[2], Henry Ho[2], Abigail Klein[2], John K. Fallon[3], Paavo Honkakoski[2,4], Kim L. R. Brouwer[2*]

[1]Department of Pharmacology, UNC School of Medicine, University of North Carolina at Chapel Hill, Chapel Hill, North Carolina, USA.

[2]Division of Pharmacotherapy and Experimental Therapeutics, UNC Eshelman School of Pharmacy, University of North Carolina, Chapel Hill, North Carolina, USA.

[3]Division of Pharmacoengineering and Molecular Pharmaceutics, UNC Eshelman School of Pharmacy, University of North Carolina, Chapel Hill, North Carolina, USA.

[4]School of Pharmacy, University of Eastern Finland, Kuopio, Finland

Supplementary Methods

Western blotting of membrane proteins was performed as described previously.[1] Samples were reduced with NuPAGE LDS sample buffer and 50 mM dithiothreitol for SDS polyacrylamide gel electrophoresis using NuPAGE 4-12% Bis-Tris gels. Protein transfer to a polyvinylidene difluoride membrane was carried out overnight at 15 V. Subsequently, the membranes were blocked in TBS-T buffer [(Tris-buffered saline) and 0.1% (v/v) Tween 20] containing 5% (w/v) nonfat dry milk. The blocked membranes were incubated overnight at 4°C with primary antibodies (Supplementary Table S2) diluted in TBS-T with 5% BSA (w/v), followed by an incubation (1 hour, room temperature) with HRP-conjugated secondary antibodies that were diluted in blocking buffer.

Immunofluorescence conditions were similar to a previous report.[2] Cells were fixed in 4% paraformaldehyde for 15 min followed by washing with phosphate buffered saline (PBS) for 5 min three times and treated with methanol for 5 min at -20°C. After washing with PBS for 5 min three times, cells were blocked and permeabilized with blocking buffer (1% BSA and 0.5% Triton X-100 in PBS) for one hour followed by an incubation with primary antibodies (Supplementary Table S2) overnight at 4°C. Then, cells were washed with PBS for 5 min three times followed by incubation with secondary antibodies for 60 min and washing with PBS for 5 min three times. After incubating with 1 μg/mL 4′,6-diamidino-2-phenylindole (DAPI) for 5 min, cells were washed with PBS for 5 min three times.

Supplementary Table S1. Gene-specific Taqman probes used for mRNA analysis.

Target gene	Taqman probe	Dye
CYP7A1	Hs00167982_m1	FAM-MGB
CYP8B1	Hs00244754_s1	FAM-MGB
CYP27A1	Hs00168003_m1	FAM-MGB
BAAT	Hs00156051_m1	FAM-MGB
BACS	Hs01556990_g1	FAM-MGB
CYP7B1	Hs01046431_m1	FAM-MGB
CYP3A4	Hs00231968_m1	FAM-MGB
ACTB	Hs01060665_g1	FAM-MGB

Supplementary Table S2. Source and dilution of primary and secondary antibodies used for immunostaining (WB) and immunofluorescence (IF) analysis.

Antibody/Protein	Source (Catalog No.)	Dilution	Analysis
BSEP	Santa Cruz Biotechnology (F-6, sc-74500)	1:200	WB
NTCP	Abcam (ab131084)	1:1,000	WB
Na^+/K^+ ATPase	Abcam (ab185065)	1:10,000	WB
OSTα	Abcam (ab103442)	1:250	WB
OSTβ	Sigma-Aldrich (HPA008533)	1:150	WB
MRP4	Cell Signaling Technology (cs12705)	1:1,000	WB
OATP1B3[3]	Custom-made; provided by Dr. Wei Yue	1:1000	WB
BSEP	Santa Cruz Biotechnology (F-6, sc-74500)	1:40	IF
MRP2	Kamiya Biomedical (MC-206)	1:25	IF
MRP3	Abcam (ab204322)	1:50	IF
MRP4	Cell Signaling Technology (cs12705)	1:100	IF
NTCP	GeneTex (GTX17693)	1:50	IF
OATP1B1	Novus Biologicals (NB100-74481)	1:50	IF
OATP1B3[3]	Custom-made; provided by Dr. Wei Yue	1:250	IF
OATP2B1	Santa Cruz Biotechnology (sc-135099)	1:100	IF
OSTα	Abcam (ab103442)	1:50	IF
OSTβ	Sigma-Aldrich (HPA008533)	1:50	IF
ZO1	Abcam (ab221547)	1:100	IF
Na^+/K^+ ATPase	Santa Cruz Biotechnology (sc-48345)	1:100	IF
Na^+/K^+ ATPase	Abcam (ab185065)	1:200	IF
Goat anti-mouse	Jackson ImmunoResearch (115-035-003)	1:10,000	WB
Goat anti-rabbit	Jackson ImmunoResearch (111-035-144)	1:10,000	WB

Supplementary Table S3. Selected signature peptides and multiple reaction monitoring (MRM) transitions for proteomic analysis of metabolic enzymes and transporters. Bold letters show amino acids labeled with stable isotope (^{13}C and ^{15}N). Trypsin does not cleave the amide bond between lysine (K) and proline (P) as highlighted in red (KP).

Protein (Gene)	Signature Peptide	MRM (Analyte; A, Internal Standard; IS)
BSEP (ABCB11)	STALQLIQ**R**	A: 515.3/529.3 (y4), 515.3/657.4 (y5); IS: 520.3/539.3 (y4), 520.3/667.4 (y5)
CES1	ELIPEATE**K**	A: 515.3/674.3 (y6), 515.3/337.7 (y6); IS: 519.3/682.4 (y6), 519.3/341.7 (y6)
CES2	TTHTGQVLGSLVHV**K**	A: 395.0/426.8 (y8), 526.3/739.4 (y7); IS: 397.0/430.8 (y8), 529.0/747.5 (y7)
CYP3A4	LSLGGLLQPEKPVVL**K**	A: 564.4/689.4 (y13), 564.4/660.9 (y12); IS: 567.0/693.4 (y13), 567.0/664.9 (y12)
CYP3A5	DTINFLSK	A: 469.3/217.1 (b2), 469.3/721.4 (y6); IS: 473.3/217.1 (b2), 473.3/729.4 (y6)
CYP2C8	NLNTTAVT**K**	A: 481.3/734.4 (y7), 481.3/620.4 (y6); IS: 485.3/742.4 (y7), 485.3/628.4 (y6)
CYP2C9	GIFPLAE**R**	A: 451.8/585.3 (y5), 451.8/293.2 (y5); IS: 456.8/595.3 (y5), 456.8/298.2 (y5)
CYP2J2	DFIDAYL**K**	A: 492.8/722.4 (y6), 492.8/609.3 (y5); IS: 496.8/730.4 (y6), 496.8/617.3 (y5)
CYP3A7	EIDTVLPN**K**	A: 514.8/358.2 (y3), 514.8/786.5 (y7); IS: 518.8/366.2 (y3), 518.8/794.5 (y7)
ENT1 (SLC29A1)	WLPSLVLA**R**	A: 527.8/755.5 (y7), 527.8/378.2 (y7); IS: 532.8/765.5 (y7), 532.8/383.3 (y7)
FMO5	IISGLV**K**	A: 365.2/503.3 (y5), 365.2/616.4 (y6); IS: 369.3/511.3 (y5), 369.3/624.4 (y6)
Gamma GTP (GGT1,2,3P)	LFQPSIQLA**R**	A: 586.8/784.5 (y7), 586.8/389.2 (b3); IS: 591.9/794.5 (y7), 591.9/389.2 (b3)
MATE1 (SLC47A1)	GGPEATLEV**R**	A: 514.8/457.8 (y8), 514.8/617.4 (y5); IS: 519.8/462.8 (y8), 519.8/627.4 (y5)
MRP2 (ABCC2)	LTIIPQDPILFSGSL**R**	A: 885.5/441.3 (b4), 885.5/989.6 (y9); IS: 890.5/441.3 (b4), 890.5/999.6 (y9)
MRP3 (ABCC3)	GALVAVVGPVGCG**K**	A: 642.4/674.3 (y7), 642.4/773.4 (y8); IS: 646.4/682.4 (y7), 646.4/781.4 (y8)
MRP4 (ABCC4)	DNEESEQPPVPGTPTL**R**	A: 622.6/371.2 (y7)/ 622.6/741.4 (y7); IS: 626.0/376.2 (y7)/ 626.0/751.4 (y7)
Na^+/K^+ ATPase (ATP1A1)	VDNSSLTGESEPQT**R**	A: 540.6/501.3 (y4), 810.4/501.3 (y4); IS: 543.9/511.3 (y4), 815.4/511.3 (y4)
OAT2 (SLC22A7)	NVALLALP**R**	A: 483.8/753.5 (y7), 483.8/569.4 (y5); IS: 488.8/763.5 (y7), 488.8/579.4 (y5)
OAT7 (SLC22A9)	DTLTLEIL**K**	A: 523.3/716.5 (y6), 523.3/829.5 (y7); IS: 527.3/724.5 (y6), 527.3/837.6 (y7)

OATP1B1 (SLCO1B1)	NVTGFFQSF**K**	A: 587.8/961.5 (y8), 587.8/860.4 (y7); IS: 591.8/969.5 (y8), 591.8/868.5 (y7)
OATP2A1 (SLCO2A1)	VNTAAVNLVPGDP**R**	A: 711.9/541.3 (y5), 474.9/541.3 (y5); IS: 716.9/551.3 (y5), 478.3/551.3 (y5)
OATP2B1 (SLCO2B1)	YYNNDLL**R**	A: 535.8/744.4 (y6), 535.8/907.5 (y7); IS: 540.8/754.4 (y6), 540.8/917.5 (y7)
OCT3 (SLC22A3)	GIALPETVDDVE**K**	A: 693.4/1031.5 (y9), 693.4/355.2 (b4); IS: 697.4/1039.5 (y9), 697.4/355.2 (b4)
P-gp (MDR1, ABCB1)	IIDNKPSIDSYS**K**	A: 493.9/627.3 (y11), 493.9/896.4 (y8); IS: 496.6/631.3 (y11), 496.6/904.5 (y8)
POR	GVATNWL**R**	A: 458.8/760.4 (y6), 458.8/588.3 (y4); IS: 463.8/770.4 (y6), 463.8/598.3 (y4)
SULT1A1	VHPEPGTWDSFLE**K**	A: 547.9/738.4 (y6), 547.9/623.3 (y5); IS: 550.6/746.4 (y6), 550.6/631.4 (y5)
SULT2A1	TLEPEELNLIL**K**	A: 706.4/534.8 (y9), 706.4/344.2 (b3); IS: 710.4/538.8 (y9), 710.4/344.2 (b3)
UGT1A10	YFSLPSVVFT**R**	A: 658.4/805.5 (y7), 658.4/1005.6 (y9); IS: 663.4/815.5 (y7), 663.4/1015.6 (y9)
UGT1A9	GILCHYLEEGAQCPAPLSYVP**R**	A: 844.1/999.6 (y9), 844.1/831.5 (y7); IS: 847.4/1009.6 (y9), 847.4/841.5 (y7)
UGT2A3	VILEELIV**R**	A: 542.3/871.5 (y7), 542.3/758.4 (y6); IS: 547.3/881.5 (y7), 547.3/768.4 (y6)
UGT2B15	FSVGYTFE**K**	A: 539.3/744.4 (y6), 539.3/843.4 (y7); IS: 543.3/752.4 (y6), 543.3/851.4 (y7)
UGT2B17	FSVGYTVE**K**	A: 515.3/795.4 (y7), 515.3/696.4 (y6); IS: 519.3/803.4 (y7), 519.3/704.4 (y6)
UGT2B4	ADIWLI**R**	A: 443.8/587.4 (y4), 443.8/486.2 (b4); IS: 448.8/597.4 (y4), 448.8/486.2 (b4)
UGT2B7	ADVWLI**R**	A: 436.8/587.4 (y4), 436.8/686.4 (y5); IS: 441.8/597.4 (y4), 441.8/696.4 (y5)

Supplementary Table S4. Multiple reaction monitoring (MRM) transitions, collision energy and source of bile acid species and internal standards (IS) measured using liquid chromatography with tandem mass spectrometry (LC-MS/MS).

Bile Acid Species	Parent ion m/z	Daughter ion m/z	Collision Energy (V)	Retention Time (min)	Source (Catalog No.)
Cholic acid (CA)	407.2	407.2/343.0	-40/-44	15.1	Sigma (1133503)
Taurocholic acid (TCA)	514.9	514.9/79.8	-62/-126	10.2	Sigma (T4009)
Glycocholic acid (GCA)	464.0	73.9	-94	12.1	Sigma (G2878)
Chenodeoxycholic acid (CDCA)	391.2	391.2	-38	17.2	Sigma (C9377)
Taurochenodeoxycholic acid (TCDCA)	498.0	498.0/80.0	-38/-130	13.3	Sigma (T6260)
Glycochenodeoxycholic acid (GCDCA)	448.0	448.0/74.0	-38/-78	15.8	Sigma (G7059)
GCDCA 3-sulfate	528.0/263.6	528.0/74.1	-38/-24	11.8	Toronto Research Chemicals (TRC; G641270)
TCA-d_5 (IS)	519.2	519.2/79.8	-62/-128	10.2	TRC (T008852)
GCA-d_5 (IS)	469.7	74.0	-94	12.1	TRC (G641357)
CDCA-d_4 (IS)	395.2	395.2	-38	17.2	TRC (C291902)
TCDCA-d_5 (IS)	503.9	80.0	-130	13.3	TRC (T008133)
GCDCA-d_7 (IS)	454.8	74.8	-38	15.8	TRC (G641257)

Supplementary Figure S1

a

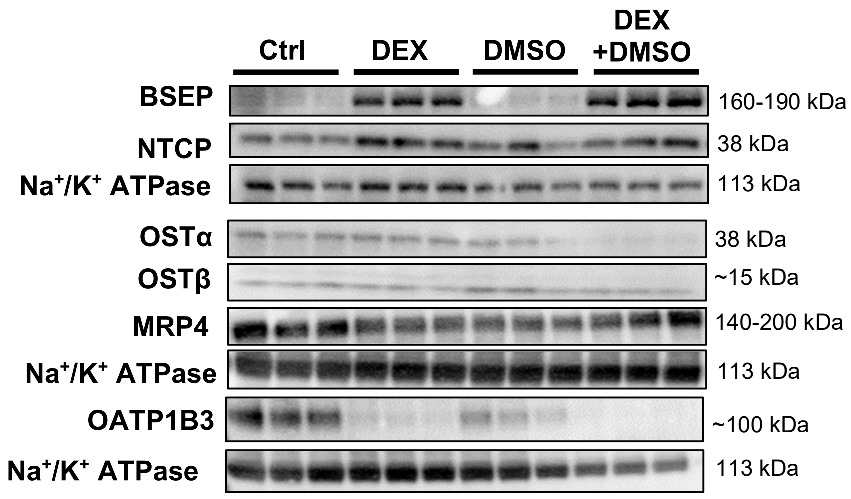

b

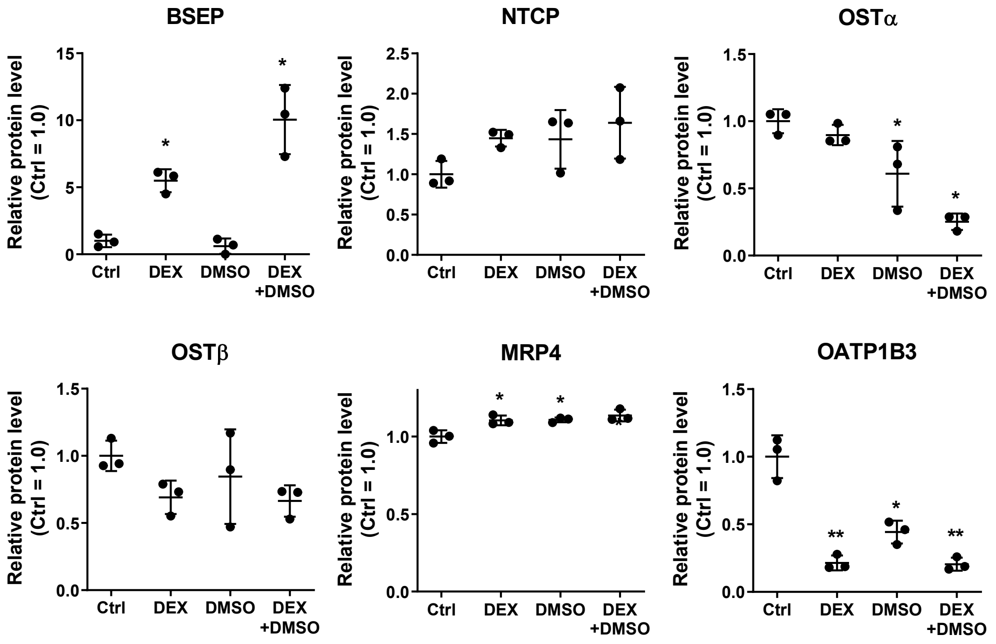

Supplementary Figure S1. Membrane protein abundance of key hepatic bile acid transporters in differentiated HuH-7 cells cultured for 2 weeks without overlay. HuH-7 cells were cultured for 2 weeks without (Ctrl) and with 1 µM dexamethasone (DEX) and/or 0.5% DMSO. **a)** Abundance of BSEP, NTCP, OSTα, OSTβ, MRP4, OATP1B3 and Na^+/K^+ ATPase (loading control) was evaluated using Western blotting of membrane fractions

harvested from HuH-7 cells without overlay. **b)** Densitometry was performed using ImageJ and BSEP, NTCP, OSTα, OSTβ, MRP4, and OATP1B3 signals were normalized to Na^+/K^+ ATPase. All treatments were performed in triplicate and relative protein levels were calculated with respect to Ctrl (set to 1.0). Statistically significant differences compared to Ctrl were assessed using an ordinary one-way ANOVA with Dunnett's multiple comparison test (*, $p<0.05$, **, $p< 0.0001$).

Supplementary Figure S2

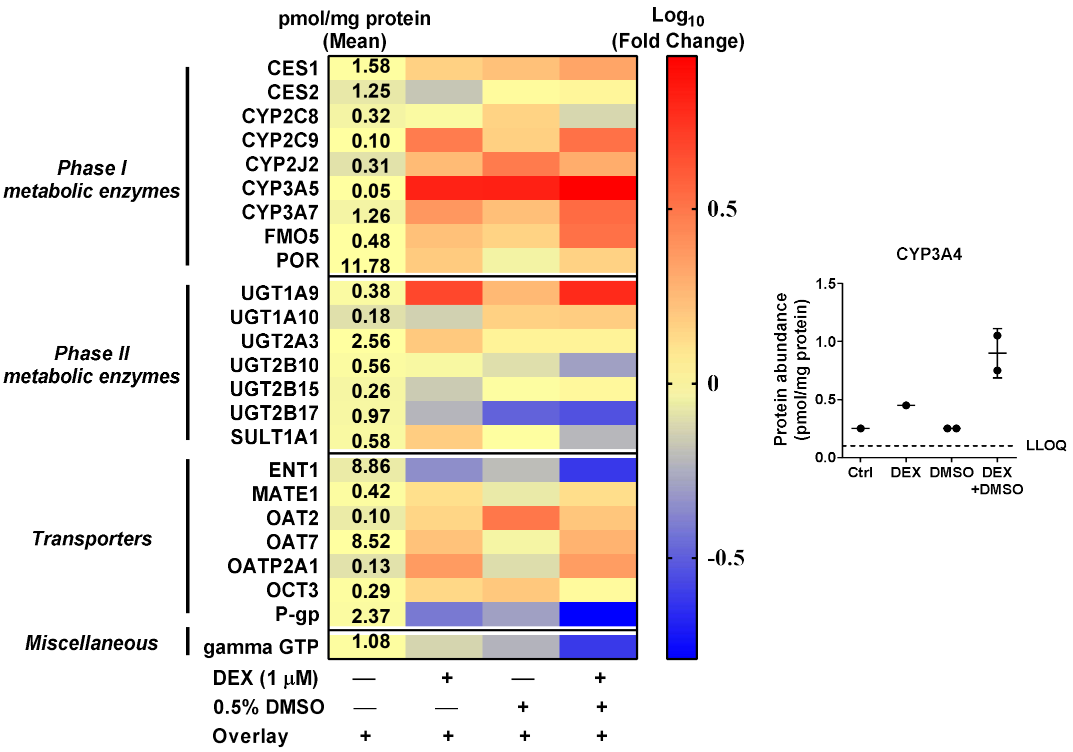

Supplementary Figure S2. Fold changes in protein abundance of metabolic enzymes and transporters in differentiated HuH-7 cells with overlay measured using proteomics. Protein abundance (pmol/mg protein) was measured in differentiated HuH-7 cells with overlay (n=3) and fold change in protein abundance was calculated with respect to control (Ctrl) HuH-7 cells with overlay and without DEX or DMSO. Log_{10} transform of fold change was plotted as a heat map (red = increase, blue = decrease). Membrane protein abundance (pmol/mg protein) of cytochrome P450 (CYP) 3A4 in 2-week cultured HuH-7 cells treated with 1 μM dexamethasone (DEX) and/or 0.5% DMSO was plotted as a scatter plot. CYP3A5 fold change was computed with the control value above the limit of detection (0.02 pmol/mg protein).

Supplementary Figure S3

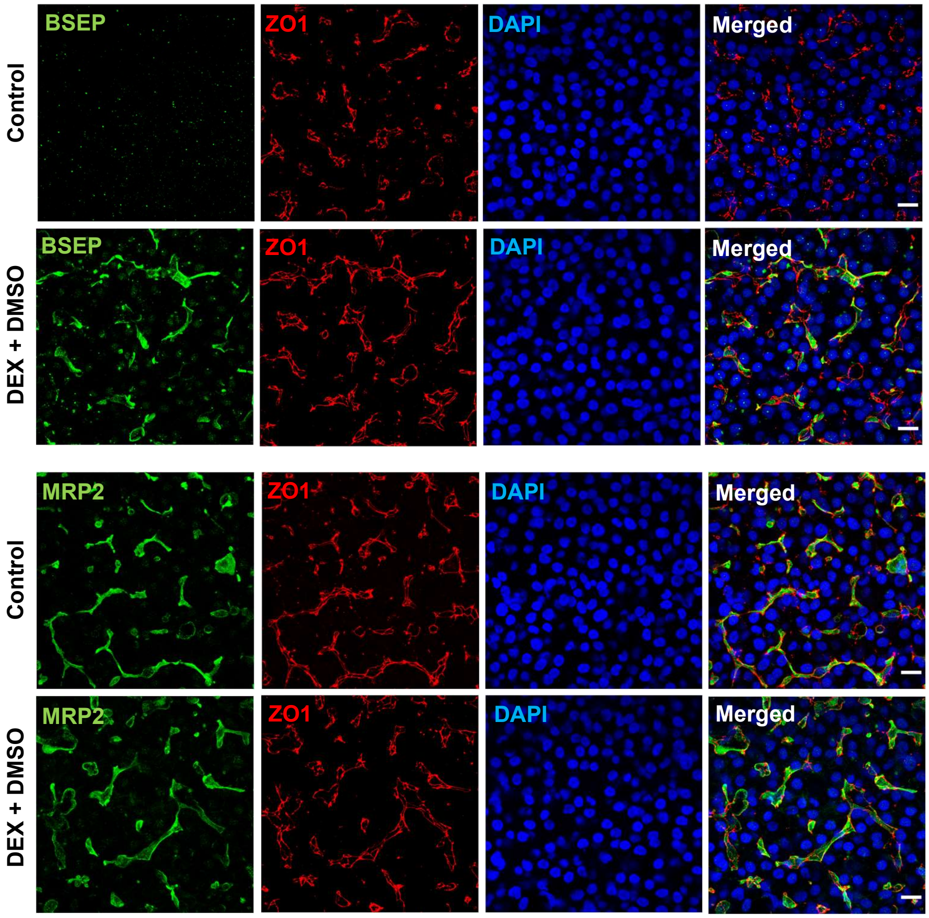

Supplementary Figure S3. Cellular localization of BSEP and MRP2 in control and differentiated (1 μM DEX+0.5% DMSO) HuH-7 cells cultured with overlay. Immunofluorescence and confocal microscopy were performed to examine localization. Immunostaining of ZO1 was used to highlight the canalicular membrane. The nucleus was stained with DAPI (Scale bar = 20 μm).

Supplementary Figure S4

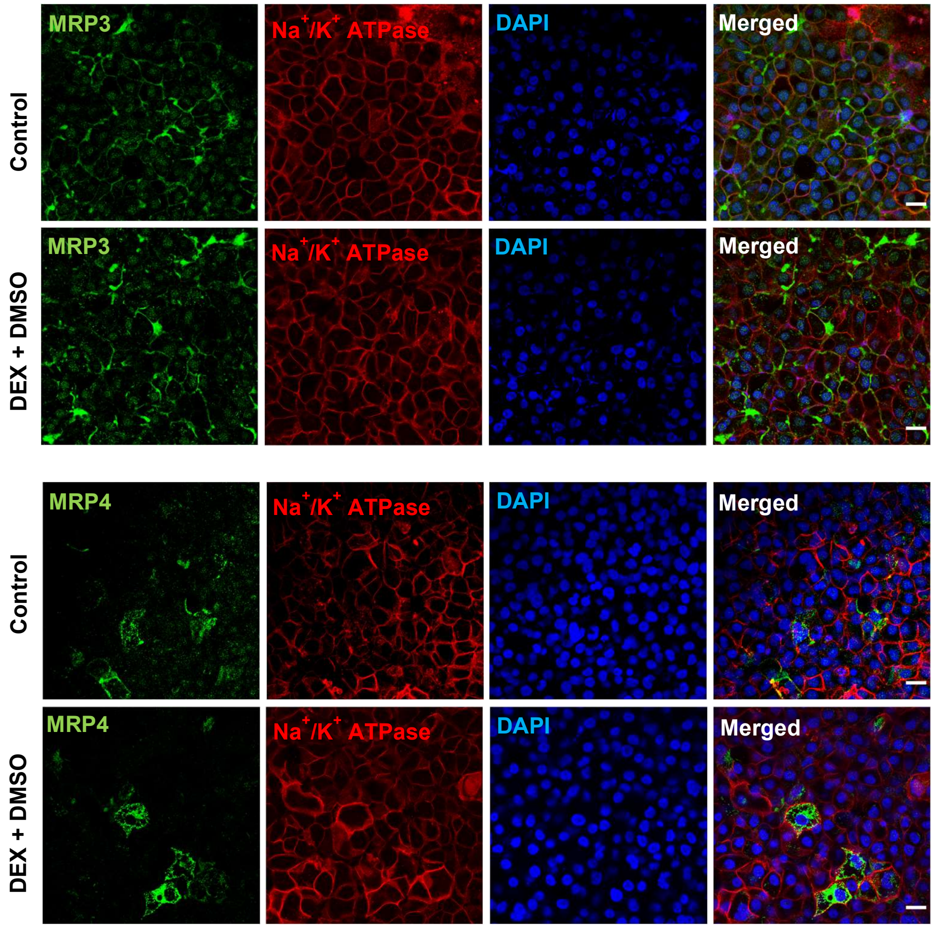

Supplementary Figure S4. Cellular localization of MRP3 and MRP4 in control and differentiated (1 μM DEX+0.5% DMSO) HuH-7 cells cultured with overlay. Immunofluorescence and confocal microscopy were performed to examine localization. Immunostaining of Na^+/K^+ ATPase was used to highlight the basolateral membrane. The nucleus was stained with DAPI (Scale bar = 20 μm).

Supplementary Figure S5

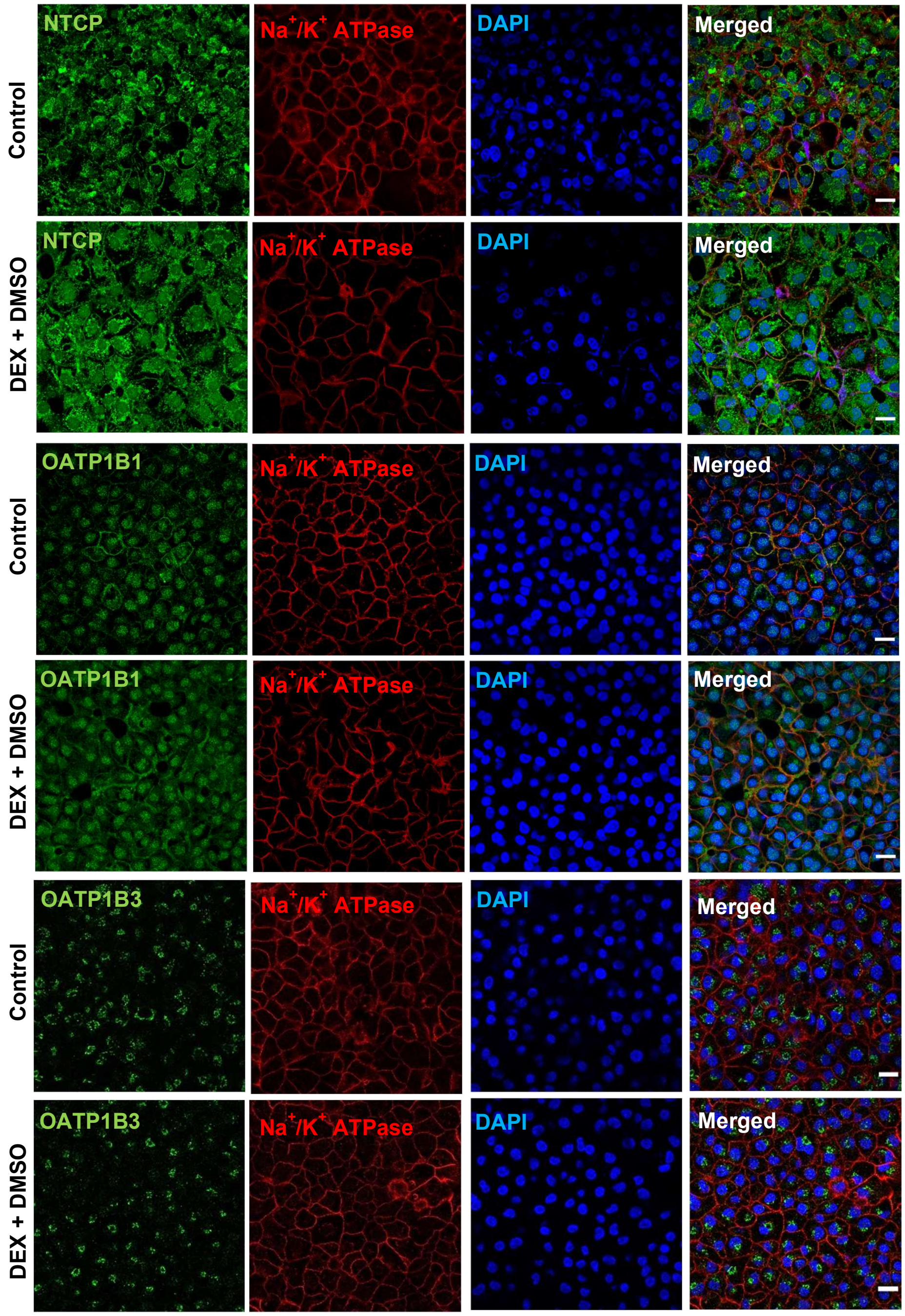

Supplementary Figure S5. Cellular localization of NTCP, OATP1B1 and OATP1B3 in control and differentiated (1 µM DEX+0.5% DMSO) HuH-7 cells cultured with overlay. Immunofluorescence and confocal microscopy were performed to examine localization. Immunostaining of Na^{+}/K^{+} ATPase was used to highlight the basolateral membrane. The nucleus was stained with DAPI (Scale bar = 20 µm).

Supplementary Figure S6

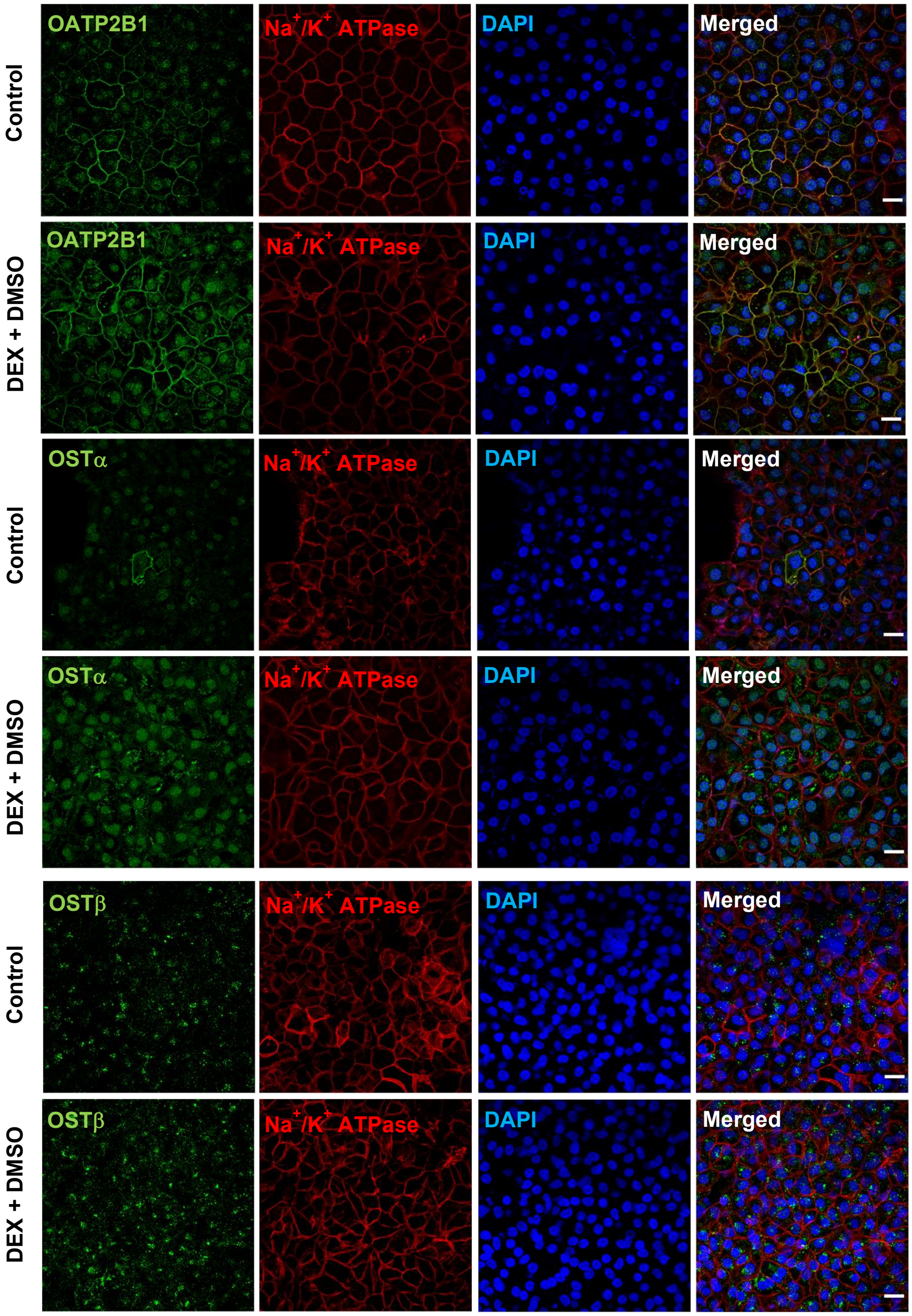

Supplementary Figure S6. Cellular localization of OATP2B1, OSTα, and OSTβ in control and differentiated (1 µM DEX+0.5% DMSO) HuH-7 cells cultured with overlay. Immunofluorescence and confocal microscopy were performed to examine localization. Immunostaining of Na^+/K^+ ATPase was used to highlight the basolateral membrane. The nucleus was stained with DAPI (Scale bar = 20 µm).

Supplementary Figure S7

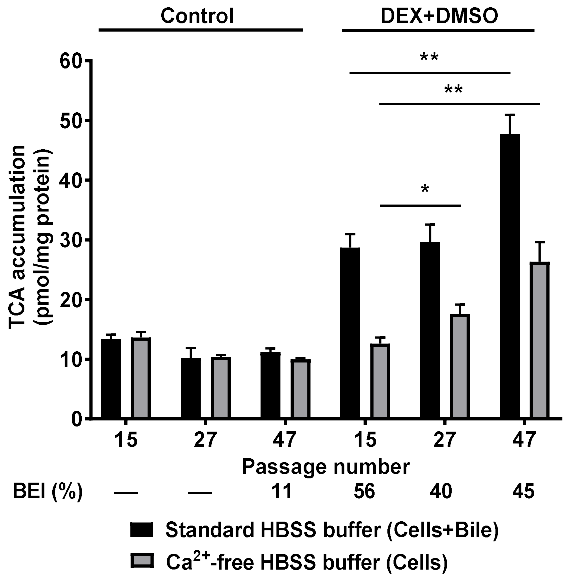

Supplementary Figure S7. Effect of cell passage number on the accumulation and biliary excretion index (BEI) of taurocholate (TCA) in control and differentiated HuH-7 cells with overlay. Accumulation and BEI of 2 μM [^{3}H]-TCA (200 nCi/mL) were measured in control (without DEX or DMSO) and differentiated HuH-7 cells with overlay in standard or Ca^{2+}-free HBSS buffer at various passage numbers; passage 15, 27, and 47. Data were plotted as mean ± standard deviation (n=3) and BEI was calculated using equation 1. Statistically significant differences were measured using an ordinary two-way ANOVA with Tukey's multiple comparisons test (*, p<0.05, **, p< 0.0001, passage 15, DEX+DMSO, versus passage 27, 47, DEX+DMSO).

References

1. Saran, C. *et al.* Novel bile acid-dependent mechanisms of hepatotoxicity associated with tyrosine kinase inhibitors. *J. Pharmacol. Exp. Ther.* **380**, 114-125 (2022).
2. Kang, S. W. S., Cogger, V. C., Le Couteur, D. G. & Fu, D. Multiple cellular pathways regulate lipid droplet homeostasis for the establishment of polarity in collagen sandwich-cultured hepatocytes. *Am. J. Physiol. Cell Physiol.* **317**, C942-C952 (2019).
3. Alam, K., Farasyn, T., Crowe, A., Ding, K. & Yue, W. Treatment with proteasome inhibitor bortezomib decreases organic anion transporting polypeptide (OATP) 1B3-mediated transport in a substrate-dependent manner. *PLoS One* **12**, e0186924 (2017).
